# Spatial predictions and uncertainties of forest carbon fluxes for carbon accounting

**DOI:** 10.1038/s41598-023-38935-8

**Published:** 2023-08-05

**Authors:** Arnan Araza, Sytze de Bruin, Lars Hein, Martin Herold

**Affiliations:** 1 Laboratory of Geo-information and Remote Sensing, Wageningen University and Research, Wageningen, The Netherlands; 2 Environmental Systems Analysis, Wageningen University and Research, Wageningen, The Netherlands; 3 Remote Sensing and Geoinformatics Section, Helmholtz GFZ German Research Centre for Geosciences, Telegrafenberg Potsdam, Germany

**Keywords:** Environmental impact, Ecosystem services

## Abstract

Countries have pledged to different national and international environmental agreements, most prominently the climate change mitigation targets of the Paris Agreement. Accounting for carbon stocks and flows (fluxes) is essential for countries that have recently adopted the United Nations System of Environmental-Economic Accounting - ecosystem accounting framework (UNSEEA) as a global statistical standard. In this paper, we analyze how spatial carbon fluxes can be used in support of the UNSEEA carbon accounts in five case countries with available in-situ data. Using global multi-date biomass map products and other remotely sensed data, we mapped the 2010–2018 carbon fluxes in Brazil, the Netherlands, the Philippines, Sweden and the USA using National Forest Inventory (NFI) and local biomass maps from airborne LiDAR as reference data. We identified areas that are unsupported by the reference data within environmental feature space (6–47% of vegetated country area); cross-validated an ensemble machine learning (RMSE=9–39 Mg C $\textrm{ha}^{-1}$ and $\textrm{R}^{2}$=0.16–0.71) used to map carbon fluxes with prediction intervals; and assessed spatially correlated residuals (<5 km) before aggregating carbon fluxes from 1-ha pixels to UNSEEA forest classes. The resulting carbon accounting tables revealed the net carbon sequestration in natural broadleaved forests. Both in plantations and in other woody vegetation ecosystems, emissions exceeded sequestration. Overall, our estimates align with FAO-Forest Resource Assessment and national studies with the largest deviations in Brazil and USA. These two countries used highly clustered reference data, where clustering caused uncertainty given the need to extrapolate to under-sampled areas. We finally provide recommendations to mitigate the effect of under-sampling and to better account for the uncertainties once carbon stocks and flows need to be aggregated in relatively smaller countries. These actions are timely given the global initiatives that aim to upscale UNSEEA carbon accounting.

## Introduction

Under the increasing threat of climate change, countries have reaffirmed the Paris Agreement commitments at the 2021 COP26 and the 2022 COP27 toward reducing ${\textrm{CO}}_{2}$ emissions and increasing ${\textrm{CO}}_{2}$ removals through forest protection and tree planting^1^. The “biocarbon” or the combined above-ground, below-ground and soil carbon of forests have contributed 23-30% of the total anthropogenic Greenhouse Gas (GHG) emissions worldwide^1,2^. To track country commitments, regular accounting of biocarbon (herein called carbon) serves as a primary source of information. Countries report their GHG inventories according to the United Nations Framework Convention on Climate Change (UNFCCC). Countries are also encouraged to develop carbon accounts under the UN System of Environmental-Economic Accounting - ecosystem accounting framework (UNSEEA), which is now an international statistical standard^3^. The UNFCCC and UNSEEA carbon accounting follow complementary measurement methods of forest carbon stocks and flows. Their quantification of flows involves both carbon emissions (stock reduction) and sequestration (stock addition). The two systems differ in the way UNSEEA accounts for all stocks and flows of carbon^3^, whereas UNFCCC focuses on reporting human-influenced emissions. Particularly, UNSEEA includes stock reductions caused by emissions due to land use and land cover (LULC) changes or natural disturbances, while carbon stock additions are mostly from tree increments due to growth. Furthermore, UNSEEA is a spatially explicit system that analyze ecosystems, where national or sub-national maps of ecosystem type, condition and ecosystem services are compiled. The UNSEEA carbon stocks and flows are commonly aggregated and reported by ecosystem type under the UN Land Cover Classification System in accounting periods of usually 1 year^3^.

Forest carbon accounting benefits from remotely sensed data^3,4^. Remote sensing data are integrated with country in-situ data such as national forest inventories (NFI) for mapping above-ground biomass maps and hence carbon stocks^5,6^. Alternatively, carbon stocks can be obtained using LULC maps and associated biomass averages^2^. The carbon flows of the carbon accounting period can also be estimated using remote sensing data. Countries rely on LULC changes and associated biomass and carbon changes to derive the gross stock reductions^7^. For gross stock additions, countries commonly use proxy indicators like Net Primary Productivity maps^8^. The gross additions are subtracted from the gross reductions to derive the closing stocks and the net ecosystem carbon balance in UNSEEA terms which is also net carbon fluxes. There are around 24 countries that have used these approaches for UNSEEA reporting^9^.

The net carbon fluxes within an accounting period (flows) can be mapped if countries have repeated in-situ data^10^. These are data from repeated NFIs and even from airborne LiDAR surveys wherein the period between the first survey and the re-measurement is the accounting period. This gives carbon estimates in two periods and hence a net carbon flux of the in-situ data. For such countries, flows of net carbon fluxes (herein called carbon fluxes) can be obtained either by direct mapping where carbon flux is the output of a mapping model or by indirect mapping where separate maps of opening and closing stocks are produced and subtracted. Most past studies favoured direct mapping to avoid propagation of mapping errors from two models^11^. Direct mapping often uses machine learning predictive models that relate in-situ biomass change to auxiliary remote sensing variables. 

Carbon accounting based on remote sensing products is affected by large uncertainty in the input data used^12,13^. The main input of carbon accounts is the carbon flux map, which tends to underestimate carbon losses owing to remote sensing signal saturation in dense forests^14^. Additionally, remote sensing signals from gradual carbon changes such as forest degradation and regrowth are most vulnerable to signal noise^15^. Forest management activities that result in tree removals such as thinning and salvage cutting can also be difficult to detect. Moreover, carbon flux maps that are derived using in-situ data can be inaccurate depending on how biomass is estimated and how plots are sampled. For instance, a clustered sample may result in preferential sampling of the spatial variability both in geographic and feature space - the latter refers to a set of environmental regions defined by the remote sensing data in relation to carbon flux. A clustered sample leads to overly optimistic accuracy estimates assessed by cross-validation^16^. Several studies analyzed the representativeness of samples in environmental feature space^17^. Such analysis can support decisions on whether to integrate additional samples to minimize the sampling uncertainty^18^ or constrain the mapping in areas where predictions are supported by the sample resulting in an incomplete map^19^.

The UNSEEA carbon accounting requires map inputs with a high spatial resolution to account for all kinds of fluxes (flows) even those from small land cover changes^7,20^. Furthermore, high-resolution maps are required for evaluating policies and implementations that concern carbon retention in ecosystems. The need for high-resolution maps may favour the use of recent global maps such as the World Resource Institute (WRI) 2000–2019 carbon fluxes at 30 m^21^ and the 100 m multi-date biomass maps (2010, 2017 and 2018) from European Space Agency Climate Change Initiative (CCI)^22^. While it is practical to use either one or create an ensemble of their carbon fluxes, they showed low to moderate agreement against in-situ data in terms of biomass change^14^. Map-based carbon fluxes are derived using either of two different methods. Subtracting the 2018 and 2010 CCI maps results in a stock difference, while the WRI follows a gain-loss method that incorporates spatial carbon emissions and removals based on activity data. Aside from global biomass maps, other remote sensing data like height, tree cover and vegetation indices may also be related to biomass change i.e. as covariates.

The main objective of this paper is to analyze how spatial carbon fluxes can be used in support of the UNSEEA carbon accounting of the above-ground carbon pool in five case countries with available in-situ data. Particularly, we use global biomass and other environmental data as covariates, together with in-situ data from NFI and local above-ground biomass maps from airborne LiDAR as reference for an ensemble machine learning framework. First, we directly map 2010 to 2018 net above-ground biomass change (living biomass) and hence derive carbon flux maps. The carbon flux map units (pixels) need to be aggregated for every forest type recommended by UNSEEA. Here we report the uncertainties associated with the spatial aggregation step (e.g. of stocks and flow residuals) as this component is outside the framework of machine learning models^23^. This uncertainty framework is also suited for net carbon fluxes instead of separately accounting for two uncertainty sources (gross stock additions and reductions) that are likely co-dependent. Lastly, we include non-forest woody vegetation in the carbon accounting. Our specific objectives are:

1. Assess to what extent spatial predictions of carbon fluxes are supported by the country data.
2. Conduct ensemble machine learning to map carbon fluxes at national scale using global biomass and other remotely sensed data as covariates.
3. Use the resulting carbon flux map to compile UNSEEA carbon accounts with reported uncertainties, identified limitations and recommendations toward upscaling to other countries.

## Methods

### Overview

**Figure 1:**
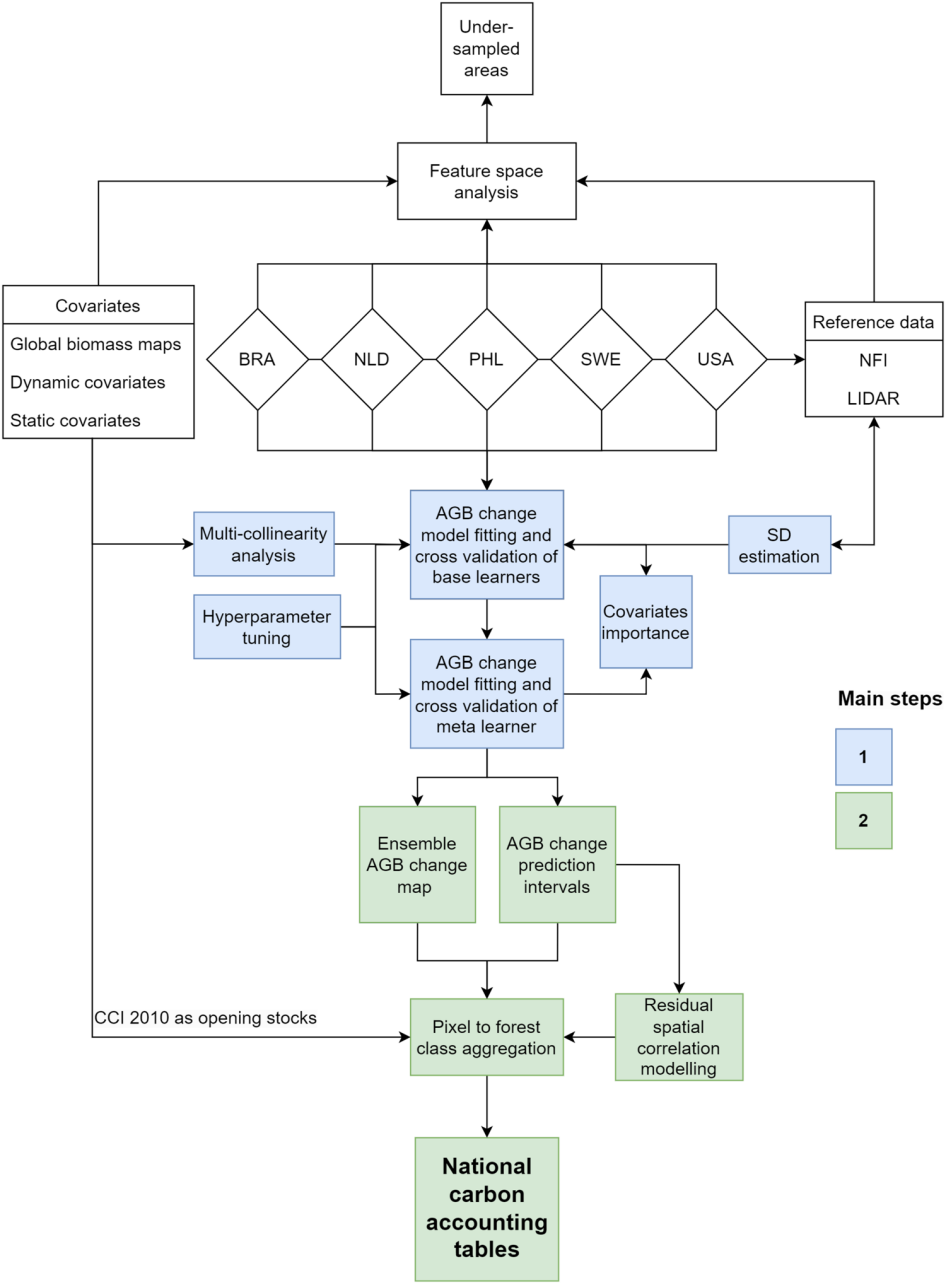
Flowchart of the steps undertaken to derive the carbon accounting tables as the final output.

The methodology overview is shown in Fig. 1, where the main steps include: (1) mapping the carbon flux using a cross-validated ensemble machine learning with remote sensing data as covariates and in-situ data (NFI or LiDAR) as reference and (2) compilation of the carbon accounts including the aggregation of the carbon flux map to each UNSEEA class. The carbon accounts represent an opening stock in 2010 derived from the CCI map 2010 and a closing stock in 2018 as the difference between the opening stock and the carbon fluxes. The overall methodology also includes intermediate steps such as estimating reference data uncertainties, investigating multi-collinearity among covariates, hyperparameter tuning of spatial models and variogram analysis. An independent step is the feature space analysis to identify areas that are not supported by the country data relative to the set of covariates used for carbon flux mapping. All steps were implemented using the *R* programming language. The feature space analysis and spatial predictions were implemented using *Slurm* high-performance computing, an open-source workload manager.

### Study area and reference data

The national ecosystem carbon accounting is demonstrated using five country cases: Brazil (BRA), the Netherlands (NLD), the Philippines (PHL), Sweden (SWE) and the United States of America (USA). The selection of countries was mainly based on reference data availability (re-measurements) and representation of major climatic zones. See Fig. 1 for maps of the study and the reference data sampling locations. The Netherlands and Sweden data are NFIs, accessed from the European NFI database with plot sizes of 0.03–0.04 ha^24^. The Philippines data is also from an NFI (0.5 ha) and was accessed directly from the Philippine environmental office. The NFI data were acquired by probability sampling: Sweden and Philippines NFIs use systematic samples while the Netherlands NFI applies stratified random sampling. The next set of reference data are high-quality local above-ground biomass maps from airborne LiDAR campaigns in Brazil^25^ and the USA^26,^, both pre-processed following the Labriere et al. 2018 methodology^27^. The USA LiDAR data also follows a systematic sample but not all sites have repeated LiDAR surveys^26^ while the Brazil LiDAR datasets are mostly sampled in secondary forests. Each LiDAR pixel (1 ha) was considered plot data equivalent to a systematic sample over footprint areas (Fig. S1). All reference datasets underwent quality filtering as elaborated in our recent study^14^. We retained only plots without forest area changes after the latest measurement and up to the 2018 map epoch. We also excluded data from LiDAR footprint edges that have partial overlap with map pixels. The number of observations are as follows: BRA=28,607, NLD=1562, PHL=587, SWE=12,887 and USA=110,939. The LiDAR reference data have associated uncertainty layers while the NFI plot measurement uncertainties were estimated using the error propagation method as described in our previous work^28^. We particularly estimated the largest plot uncertainty contributor originating from tree measurement errors and the use of allometric models in estimating biomass. Finally, we converted the biomass change of all reference data to carbon flux (Mg C $\textrm{ha}^{-1}$) using a 0.49 multiplier. See Table S1 and Fig. S1 for the technical details of the reference datasets.

**Figure 2:**
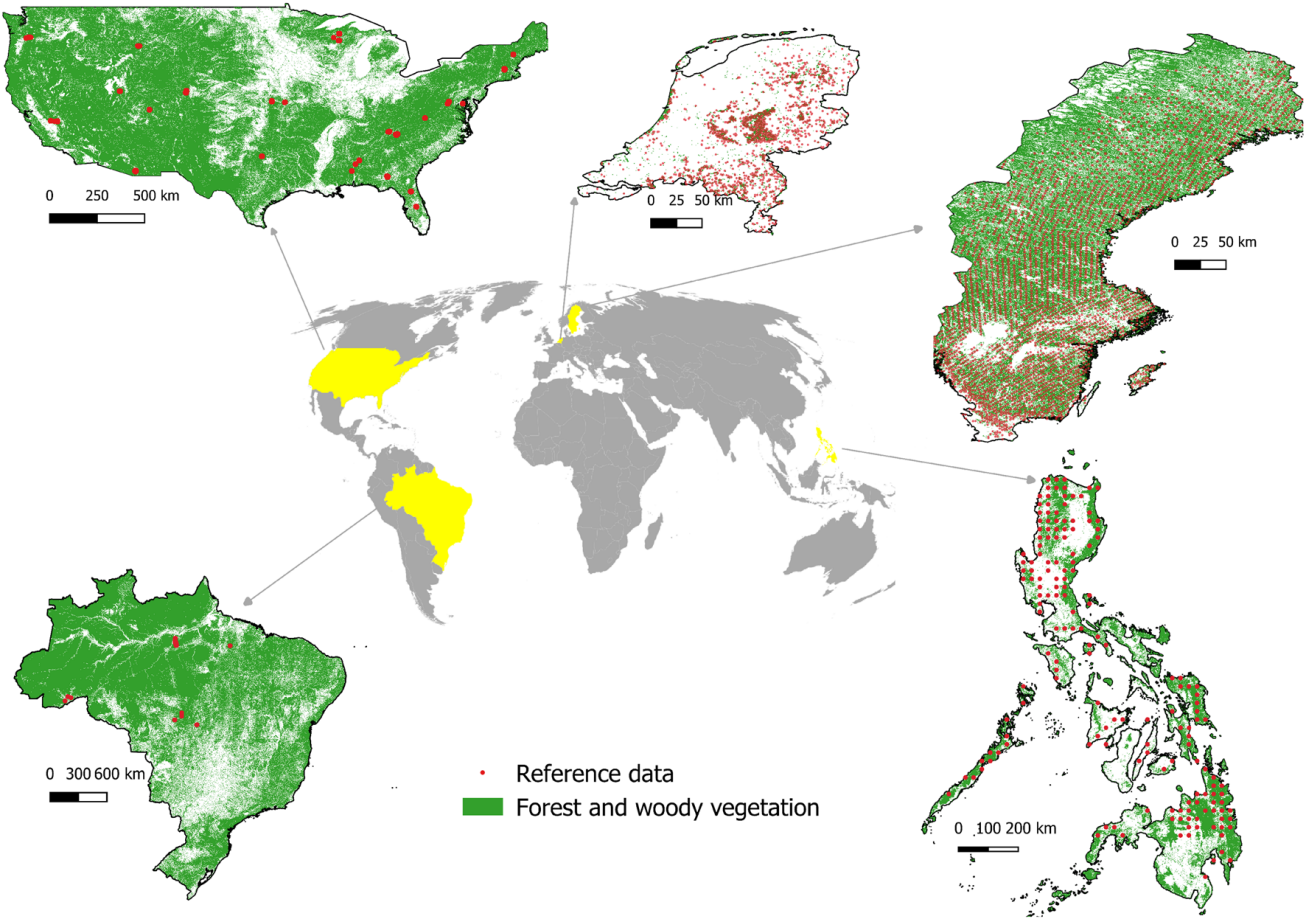
Country cases and the sampling of reference data within the forest and other woody vegetation described in Table 2 and Fig. S2. Note that countries are depicted at different scales. Note also that the USA and Brazil are LiDAR footprints where observations are BRA=28,607 and USA=110,939; see Fig. S1. We used QGIS 3.4.3^29^ to layout this map.

### Spatial covariates

The list of spatially exhaustive covariates used and their description are shown in Table 1. The first set of covariates includes global biomass maps derived from the ESA and WRI sources herein called CCI maps and WRI Flux, respectively. The CCI map was produced using radar remote sensing where biomass was retrieved from backscatter values using a semi-mechanistic model that does not rely upon plot calibration data^30^. The WRI map was an output of the modified carbon flux model of WRI that used CCI 2010 as the baseline biomass^14^. The local variability of biomass changes from these high-resolution inputs was also considered. We computed their textural variables using Gray-Level Co-Occurrence Matrix (GLCM^31^), particularly the mean, variance, homogeneity and contrast of biomass changes in a 300 x 300 m window. We also used a coarse time series biomass (JPL^32^) and a periodic biomass dataset (CONUS^33^, USA only). All biomass maps are produced without the use of our reference data. The next covariate was forest height dynamics which was obtained from height data by integrating Landsat data with Global Ecosystem Dynamics Investigation (GEDI) data^34^. We also used tree cover dynamics from multi-date tree cover datasets derived from optical satellite data^35,36^. Datasets that represent forest management^37,38^ and land cover changes^35^ were also included. For European countries, we included gap-filled quarterly composites of Normalized Vegetation Difference Index (NDVI^39^), being readily available and cloud-free i.e. never been reproduced for large and tropical countries. The remaining covariates were static variables such as forest type, elevation, slope and biomes. In total, we started with 28 covariates for European countries, 25 for the USA and 24 for Brazil and the Philippines - all from open-access data. We finally constrained all covariates within the recommended classes by UNSEEA as described in Table 2 and Fig. S2.

All covariates were cross-correlated to assess multicollinearity among them as a precaution to avoid model overfitting and misinterpretation of the covariates model importance (see Fig. S3). We randomly sampled 5000 pixels for each country for the cross-correlation. We found only a few covariate pairs that exhibit a strong correlation (> 0.8 *r*), particularly pairs that include global biomass maps and an associated textural property (*texMean* and *texVar*). We excluded the latter since they also highly correlate with other map textural properties. An exception to this step is the 2010 and 2018 land cover layers to retain a dynamic input similar to the biomass 2010 and 2018 variables.

**Table 1:** Spatial data of above-ground biomass (AGB) and other remotely sensed data used as covariates and their technical details.

Spatial data	Category	Covariates name and definition	Units	Country used	Pixel size	Epochs used	RS and in-situ data
CCI^22^	CCI	*agb10_cci* = CCI AGB 2010	Mg ha $^{-1}$	All	100 m	2010–2018	ALOS2-PALSAR2, Sentinel 1
		*agb18_cci* = CCI AGB 2018	Mg ha $^{-1}$				
		*diff_cci* = CCI biomass change	Mg ha $^{-1}$				
		*texContrast_cci* = Textural contrast of *diff_cci*	Relative				
		*texHomo_cci* = Textural homogeneity of *diff_cci*	Relative				
		*texMean_cci* = Textural mean of *diff_cci*	Relative				
		*texVar_cci* = Textural variance of *diff_cci*	Relative				
WRI^21^	Flux	*diff_flux* = WRI Flux biomass change	Mg ha $^{-1}$	All	100 m	2010–2019	CCI 2010, Global Forest Change (GFC) and IPCC activity data
		*texContrast_flux* = Textural contrast of *diff_flux*	Relative				
		*texHomo_flux* = Textural homogeneity of *diff_flux*	Relative				
		*texMean_flux* = Textural mean of *diff_flux*	Relative				
		*texVar_flux* = Textural variance of *diff_flux*	Relative				
JPL^32^	AGB	*diff_jpl*= JPL biomass change	Mg ha $^{-1}$	All	10 km	2010–2019	MODIS, ICESat, ALOS-PALSAR
CONUS^33^	CONUS	*diff_conus* = CONUS biomass change	Mg ha $^{-1}$	All	100 m	2010–2017	ALOS2-PALSAR2, MODIS
Forest height loss^34^	Environmental (envi)	*height_envi* = forest height loss	Meters	All	30 m	2010–2020	Landsat, GEDI
Forest dynamics^34^	Environmental	*forType_envi* = forest dynamics	Categorical	All	30 m	2010–2020	Landsat, GEDI
VCF dynamics^36^	Environmental	*tc_envi* = tree cover change	% cover change	SWE, NLD	30 m	2010–2015	Landsat 5, 7
NDVI quarterly composites^39^	Environmental	*ndviQ1_envi* = 1st quarter NDVI change composites	-1 to 1	SWE, NLD	30 m	2010–2019	Landsat archive
		*ndviQ2_envi* = 2nd quarter NDVI change composites	− 1 to 1				
		*ndviQ3_envi *= 3rd quarter NDVI change composites	− 1 to 1				
		*ndviQ4_envi* = 4th quarter NDVI change composites	− 1 to 1				
Land cover change^35^	Land Cover (LC)	*lcov10* = CCI Land Cover 2010	Categorical	All	300 m	2010–2019	MERIS
		*lcov18* = CCI Land Cover 2018	Categorical				
		*lcovDiff* = Land cover change	Categorical				
Forest Management^38^	Forest management (mgmt)	*mgmt_mgmt* = 2015 forest management	Categorical	All	100 m	2015	PROBA-V
Forest Landscape Integrity Index^37^	Forest management	*flii_mgmt* = Relative forest integrity index	0–100	All	300 m	2019	GFC data; Curtis et al., data
Elevation^40^	Topography (topo)	*elev_topo* = Elevation	Meters	All	30 m	2000	SRTM
Slope^40^	Topo	*slope_topo* = Slope	Degrees	All	30 m	2000	SRTM
Biome^41^	Climate	*bio_climate* = Major climatic zones	Categorical	All	10 km	2017	Protected area network

Note that the “Epochs used” column also denotes whether a covariate is dynamic or static.

### Feature space representation of reference data

This step is needed to assess how the reference data represent the spatial variability of feature space, which is becoming a prerequisite for spatial prediction models. This analysis identifies potentially under-sampled areas or those areas that exhibit high dissimilarity based on distances from the reference data in the multidimensional feature space^19^. The latter is spanned by the covariates listed in Table 1 that are used in a spatially cross-validated model to calculate dissimilarity. The non-covered areas are prone to extrapolation of the machine learning models where less credible spatial predictions are expected. Awareness of the extent of these non-covered areas is crucial not only for our carbon accounting demonstration but also as decision-support towards improving the current accounts i.e. the need for additional samples and forest masking. Here we identified under-sampled areas countrywide and for each UNSEEA carbon accounting class in Table 2 and Fig. S2.

### Prediction models of carbon fluxes

An ensemble model of three machine learning models was developed under a model generalization framework^42^. The main idea of the framework is that a generic model (meta learner) fitted from predictions of individual models (base learners) has superior predictive performance than individual predictions. Models of random forest (RFM), extreme gradient boosting and support vector machine served as base learners where each model resulted in their own predictions of carbon flux. These predictions were then used as covariates to a meta learner (RFM). Any RFM implementation starts by bootstrapping the reference data and these resamples are used to create regression trees. Through bagging the data and sub-sampling the candidate covariates at each split, the trees are aimed to be mutually uncorrelated. The random forest algorithm averages predictions over all trees to produce a final prediction. To estimate the uncertainty of the carbon flux predicted by the RFM meta learner, we used quantile regression forest where decision trees are trained on different quantile levels of the carbon flux and derive an entire distribution of carbon flux predictions^43^. We particularly used the $5\mathrm{{th}}$ and $95\mathrm{{th}}$ quantiles as prediction intervals and observed their spatial patterns and magnitude among countries. We employed case weights using inverse variance weighting to give preference to less uncertain reference data in the random forest bootstrapping procedure. The descriptions of other individual ML models are provided in the supplementary materials.

All base learners underwent model tuning whereas models that use unique combinations of hyperparameters were iterated using grid search approach. The combinations are obtained from a user-defined range of values for each hyperparameter and their unique combinations define the number of iterations. We pre-defined this range of values for RFM and support vector learners, while we mostly followed Li et al.^44^ for the extreme gradient boosting learner given that such learner is generally challenging to tune. The optimal hyperparameters combination was determined for the iteration that depicts the lowest objective function i.e. Root Mean Square Error (RMSE). See Table S2 for the final hyperparameters for each country case.

### Model evaluation

A five-fold random cross-validation was applied using all reference data to evaluate model performance. Independent folds were used by the base learners and the meta learner to avoid overfitting the latter. Denoting carbon flux as *z* at location $s_i$, three metrics were used to evaluate the models: RMSE, which is the squared difference between population means of reference data $z(s_i)$ and predictions ${\hat{z}}(s_i)$; Mean Error (ME) or the mean difference between $z(s_i)$ and ${\hat{z}}(s_i)$; and coefficient of determination ($\textrm{R}^{2}$) as the goodness of fit measure.

1 $$\begin{aligned} R M S E= & {} \sqrt{\frac{1}{n} \sum _{i=1}^n\left( z\left( s_i\right) -{\hat{z}}\left( s_i\right) \right) ^2}, \end{aligned}$$

2 $$\begin{aligned} M E= & {} \frac{1}{n} \sum _{i=1}^n z\left( s_i\right) -{\hat{z}}\left( s_i\right), \end{aligned}$$

3 $$\begin{aligned} R^2= & {} 1-\frac{\sum _{i=1}^n\left( z\left( s_i\right) -{\hat{z}}\left( s_i\right) \right) ^2}{\left. \sum _{i=1}^n\left( z\left( s_i\right) -{\bar{z}}\right) \right) ^2}. \end{aligned}$$

### Influence of covariates on models

The importance values based on covariate data permutation were quantified for each base learner. The RMSE before and after the permutation is computed and the increase in RMSE indicates the covariate importance. This comparison was done for each fold of the cross-validation and then averaged among the folds. The final importance values we used were weighted averages based on the importance score of base learners to the meta learner. The resulting importance scores were first presented individually for each covariate per country. Second, the importance values were ranked and averaged with respect to their data categories (Table 1). The second set of results is helpful to highlight the influence of global biomass data in mapping carbon fluxes. All covariates importance values range from 0 to 100%.

### Carbon accounting tables and their uncertainties

We first derived the carbon flux standard deviation $SD_{flux}$ from the prediction interval, particularly the $95\mathrm{{th}}$ quantile (Q) and $5\mathrm{{th}}$ Q limits for a 90% prediction interval:

4 $$\begin{aligned} \text {SD}_\text {flux}=\frac{{95^{th}Q} - {{5^{th}Q}}}{2} \times 1.64, \end{aligned}$$

Then, the 100 m carbon flux maps needed aggregation for every UNSEEA class in Table 2. Aside from the carbon flux, the associated uncertainties also need to be aggregated while taking into account the covariance of map errors. Moreover, the variance of map errors may vary over space i.e. owing to heteroscedasticity. Hence, we modelled the spatial correlation of the carbon flux map standardized residuals at reference data locations using variograms. We also did the same procedure for the opening stocks interchanging $SD_{flux}$ with the SD layer of CCI 2010 $SD_{open}$. The full details of this step including the standardization of carbon flux residuals are shown in the supplementary materials.

We derived the covariances of the map error component $\sigma _{{i,j}}$ of pixel pairs *i* and *j* (1...n), Eq. 5. The covariances were aggregated for each UNSEEA class to derive the variance and hence the SD of each class $SD_{fc}$ (Eq. 5). This applies to both $SD_{open}$ and $SD_{flux}$. The resulting aggregated variance of the opening stock and fluxes were added to obtain the closing stock variance assuming error independence among the two random variables. We finally took the square root of the aggregated variances of the opening stocks, fluxes and closing stocks, and reported them in the carbon accounts.

5 $$\begin{aligned} \text {SD}_{fc}=\sqrt{\sum _{i=1}^{n} \sum _{j=1}^{n} \sigma _{i, j}}. \end{aligned}$$

The minimum requirement to define the UNSEEA carbon accounting classes is land cover inputs that distinguish broadleaved, coniferous, mixed and mangrove forests (Level 2 classes). Areas with dominant and partial woody grassland, shrublands and scrubs were included and aggregated into one UNSEEA class “Other woody vegetation”, see Table S3 for the reclassification details. Multiple continental to global land cover data exist and we preferred the dataset with higher resolution and accuracy. A forest plantation dataset^38^ was integrated into the land cover maps to distinguish between natural forests and plantations since the latter is required by UNSEEA. The land cover inputs were resampled to 100 m using nearest neighbor interpolation. Then, the land cover and carbon stocks and flows inputs were projected into an equal area projection to avoid geographic area distortion especially in places far from the equator. Table 2 shows further details about the carbon accounting data inputs.

We then derived the total country net carbon fluxes and inter-compared them with similar estimates from other sources using forest area in 2010. We also reported the net emissions and net removals separately as supplementary results in Table S4. The terms we used were “net” emissions and removals since we used the net carbon flux as input. The net emissions and net sequestration were calculated as the sum of all negative and positive carbon flux pixels for each UNSEEA class, respectively. The extended table also included the net emissions driven by land-use changes (forest conversions) and emissions within forests (forest degradation) based on the periodic land cover dataset in Table 2. Net emissions in areas classified as forest both in the 2010 and 2018 land cover data were attributed to forest degradation, while net emissions in areas that are forest in 2010 but non-forest in 2018 were attributed to forest conversion.

**Table 2:** Details of the datasets used for the UNSEEA recommended carbon accounting classes.

Country	LC dataset	UNSEEA classes	Pixel size (m)
Brazil	CCI-LC^35^	Broadleaved forest, coniferous forest, mixed forest, mangroves, other woody vegetation, forest plantations	300
Netherlands	CORINE^45^	Broadleaved forest, coniferous forest, mixed forest, other woody vegetation, forest plantations	100
Philippines	CCI-LC^35^	Broadleaved forest, coniferous forest, mixed forest, mangroves, Other woody vegetation, Forest plantations	300
Sweden	CORINE^45^	Broadleaved forest, coniferous forest, mixed forest, other woody vegetation, forest plantations	100
USA	CCI-LC^35^	Broadleaved forest, coniferous forest, mixed forest, other woody vegetation, forest plantations	300

The forest plantation class was derived by integrating a plantation forest dataset^38^.

## Results

### Under-sampled areas

**Figure 3:**
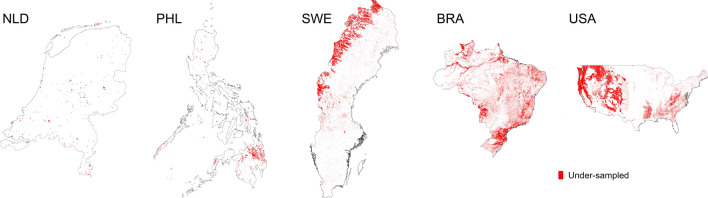
Maps showing how the current reference data represents environmental feature space. We used *ggplot2* in *R*^46^ to layout this map.

Maps of under-sampled areas according to feature space coverage are shown in Fig. 3. Notice the large extent of these areas are for Brazil and the USA. The majority of southeastern Brazil and western USA are not supported by the current sample. Per country, the relative spatial extent of non-covered areas is: NLD=6%, PHL=7%, SWE=14% BRA=4 2%, and USA=47% of the total vegetated areas of these countries. The breakdown of the non-covered areas for each UNSEEA carbon accounting class is shown in Fig. S4. The classes with the highest proportion of under-sampled areas (>50% of total class area) are mostly found in Brazil and USA, particularly coniferous, mixed, other woody vegetation and mangroves (Brazil only) forests, see Fig. S4. Among all countries, the class with the largest proportion of areas unsupported by the sample is other woody vegetation. This reflects the fact that the NFI and LiDAR reference data are mainly forest samples.

### Cross-validation of spatial models

The model evaluation results using five-fold cross-validation for each country are shown in Table 3. Overall, the cross-validation range are: RMSE=9–39 Mg C $\textrm{ha}^{-1}$, ME=− 0.3–0.2 Mg C $\textrm{ha}^{-1}$ and $\textrm{R}^{2}$=0.16-0.71. The cross-validation results differ between countries. The Netherlands and the Philippines have different results than the other countries notable in the standard deviations of the three accuracy metrics. Brazil and USA show almost similar results for every validation fold and even show the best fit with reference data. These countries used LiDAR maps as reference, but recall also that such samples are clustered (LiDAR footprints) and may produce conservative cross-validation results.

**Table 3:** Accuracy metrics of the cross-validated spatial models of carbon fluxes and the standard deviation over the five-fold cross-validation.

Country	RMSE Mg C $\textrm{ha}^{-1}$	ME Mg C ${\textrm{ha}}^{-1}$	$\textrm{R}^{2}$
Brazil	9.3 ± 0.4	0.1 ± 0.1	0.71 ± 0.02
Netherlands	23.9 ± 4.6	0.1 ± 2.1	0.21 ± 0.10
Philippines	39.4 ± 7.7	− 0.3 ± 5.5	0.16 ±0.21
Sweden	18.6 ± 0.5	0.2 ± 0.4	0.34 ± 0.01
USA	11.8 ± 0.5	− 0.1 ± 0.5	0.63 ± 0.02

### Covariates importance

**Figure 4:**
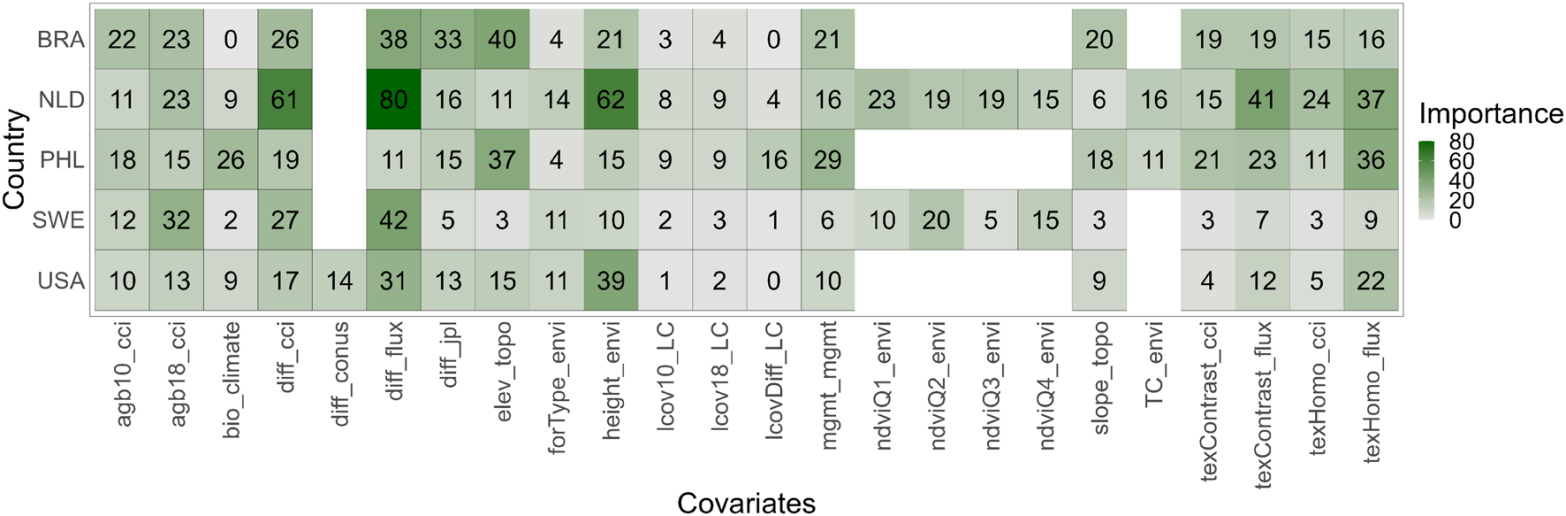
Permutation-based importance values (0–100%) of each covariate to country spatial models. Refer to Table 1 for the covariate names.

**Figure 5:**
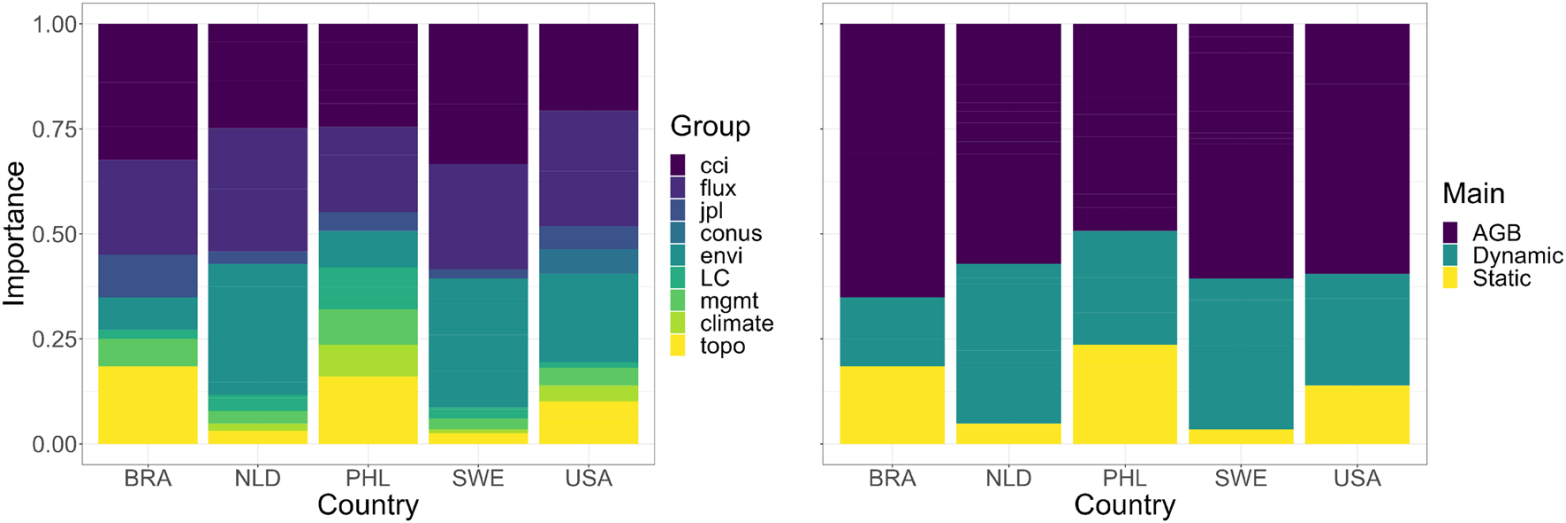
Proportion of the importance values (0–1) for each covariate group shown in Table 1. The left graph shows the category of covariates that distinguish each biomass global product along with other dynamic environmental inputs (height, tree cover and NDVI), land cover, management, climate and topography. The right graph further generalizes the covariates into categories that distinguish global above-ground biomass maps from other dynamic covariates, along with the static covariates.

The weighted importance values of the ensemble learner are shown in Fig. 4. The colored matrix shows the importance of individual covariates where models of most countries are highly influenced by the WRI Flux, CCI maps and canopy height changes. Textural variables show moderate influence on the models and quarterly NDVI to the models of European countries. The static covariates and even dynamic LC data show the least influence on most models. An exception to this is the high influence of elevation on the models of tropical countries Brazil and the Philippines. Using the mean importance of each covariate, the categorical importance values further highlight the influence of global above-ground biomass maps (bar graphs of Fig. 5). This influence accounts for 51–70% of the importance scores of covariates.

### Map predictions

**Figure 6:**
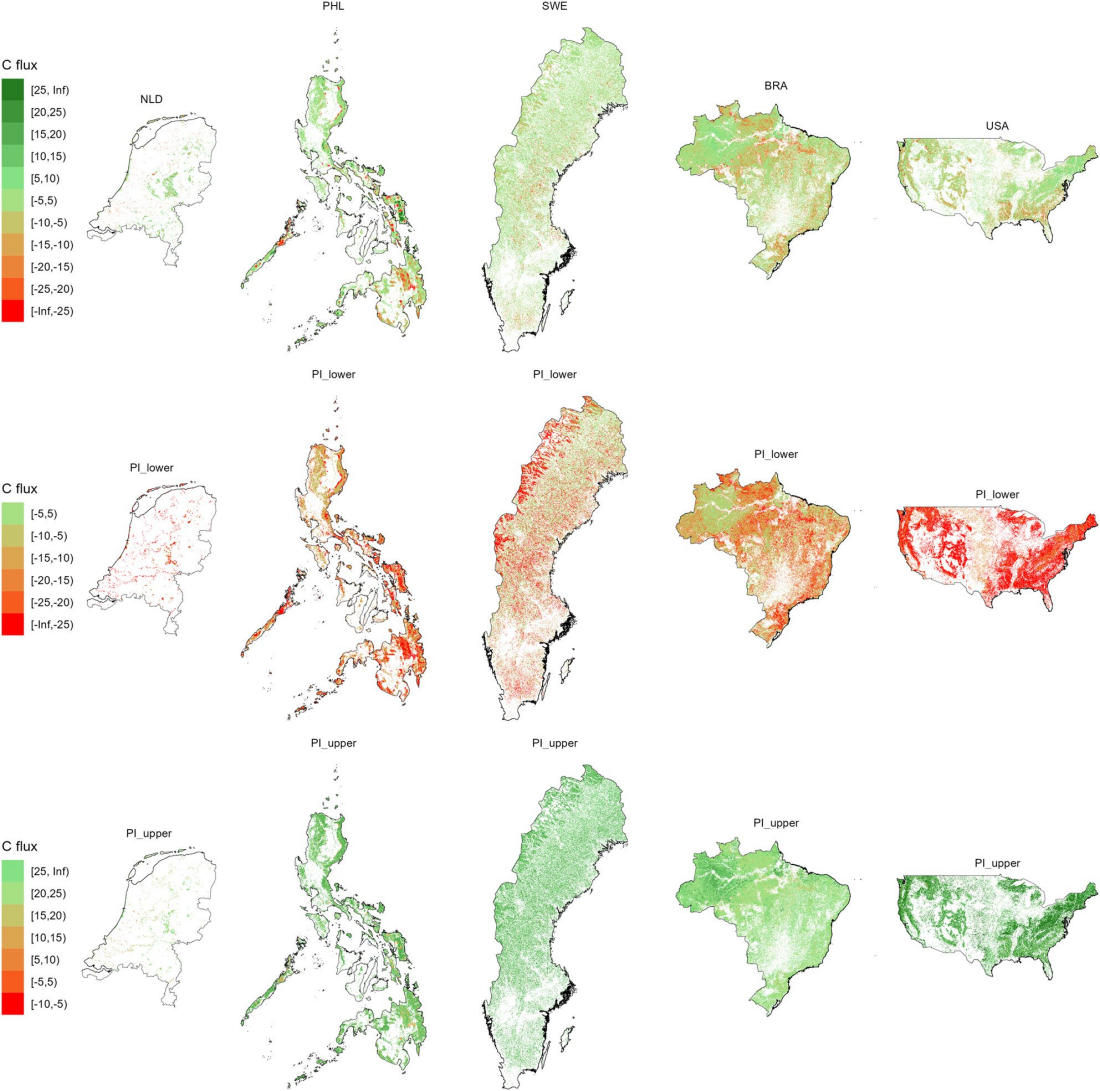
Predicted 2010–2018 carbon flux maps for all countries and their prediction intervals. Map units are Mg C $\textrm{ha}^{-1}$. Note that colors can be different between the prediction and prediction intervals. We used *ggplot2* in *R*^46^ to layout this map.

The predicted carbon flux maps and their prediction intervals are shown in Fig. 6. Spatial patterns of the past-decade carbon dynamics can be observed. Areas with evident carbon losses exist in all countries except the Netherlands. Evident also are losses that appear as regional hotspots particularly southern Philippines, central Brazil and southwest USA. The hotspots are less pronounced for Netherlands and Sweden. Forest carbon sequestrations are also observed in all countries. Most are small carbon sequestrations of around 5–25 Mg C $\textrm{ha}^{-1}$, most evident in the Amazon basin in Brazil. Such small carbon sequestration in 8 years is normal for an old-growth intact forest (Amazon) while the other cases can be attributed to natural disturbances that hinder tree growth like droughts. The loss and sequestration spatial patterns are also pronounced in the prediction intervals. The highest uncertainties of the predictions are observed in the USA map.

### Spatial aggregation and carbon accounts

The spatial correlation of carbon flux residuals depicted by the variograms is shown in Fig. S6. Spatially correlated residuals are evident in relatively short distances of 100–5500 m. The variability depicted by variogram sills ranges from 0.32–0.63. For the 2010 carbon stocks, a similar short-distance correlation of residuals is observed but in a slightly wider range and higher sill than the carbon flux. These results are the basis to ignore spatially correlated residuals when aggregating map errors for each UNSEEA class.

The resulting carbon accounting table is shown in Table 4, while the table where emissions are further disaggregated is shown in Table S4. Net fluxes of carbon emissions in 8 years are most evident and highest in number in Brazil and USA, particularly other woody vegetation of at least 95.23±11.07 Tg and plantations of at least 239.2±7.46 Tg. All other countries mostly depict net carbon sequestrations in 8 years notably USA broadleaved = 188.01±6.3 Tg, Sweden coniferous = 5.59±0.84 Tg and Philippines broadleaved = 3.87±2.57 Tg. The natural forests that consist of broadleaved, coniferous, mixed and mangroves are showing more net carbon sequestrations than plantations except in Brazil where all classes are found as carbon emitters mainly due to land use conversion in these forests in the period 2010 to 2018. The observation in Brazil is also consistent with the reports from FRA (see Table S5). Except for Brazil, natural broadleaved forest shows net carbon sequestration. Table S4 shows that most emissions are driven by land-use changes in all countries (areas classified as forest in 2010 and non-forest in 2018), while the net emissions within forest areas (areas classified as forests in 2010 and 2018 land cover data) are minimal. The uncertainty estimates of carbon fluxes over 8 years are generally higher than the stocks, while the closing stocks are more uncertain than the opening stocks.

**Table 4:** Carbon accounting tables for 2010–2018 of the five case countries.

UNSEEA class	Area (’000 $\textrm{km}^{2}$)	Opening 2010 (Tg C)	Net fluxes (Tg C)	Closing 2018 (Tg C)
BRA				
Broadleaved	3485634	49541.1 ± 30.175	– 43.18 ± 4.961	49497.92 ± 29.765
Coniferous	153277	1059.03 ± 3.905	– 22.76 ± 1.083	1036.27 ± 3.752
Mixed	735145	1988.52 ± 5.121	– 156.02 ± 2.390	1832.50 ± 4.529
Mangroves	166677	794.79 ± 3.496	– 0.48 ± 1.010	794.31 ± 3.347
Other woody vegetation	1140095	4015.19 ± 8.232	– 403.50 ± 3.069	3611.69 ± 7.638
Plantations	1320002	3380.46 ± 6.601	– 421.18 ± 3.112	2959.28 ± 5.822
NLD				
Broadleaved	3008	0.33 ± 0.064	0.002 ± 0.030	0.33 ± 0.057
Coniferous	4962	0.25 ± 0.082	0.001 ± 0.015	0.25 ± 0.081
Mixed	5487	2.2 ± 0.164	0.02 ± 0.033	2.22 ± 0.161
Other woody vegetation	54494	1.93 ± 0.253	0.02 ± 0.214	1.95 ± 0.135
Plantations	15199	5.41 ± 0.254	0.02 ± 0.063	5.43 ± 0.247
PHL				
Broadleaved	57334	427.45 ± 4.060	3.87 ± 2.476	431.32 ± 3.218
Coniferous	3008	14.42 ± 0.669	0.14 ± 0.457	14.56 ± 0.488
Mixed	4962	12.19 ± 0.659	– 0.28 ± 0.511	11.91 ± 0.416
Mangroves	5487	7.71 ± 0.640	– 1.10 ± 0.583	6.61 ± 0.263
Other woody vegetation	54494	297.88 ± 3.191	1.83 ± 1.770	299.71 ± 2.654
Plantations	15199	45.6 ± 1.280	– 0.86 ± 0.858	44.74 ± 0.949
SWE				
Broadleaved	4588	13.26 ± 0.254	0.32 ± 0.114	13.58 ± 0.227
Coniferous	109355	501.12 ± 2.169	5.59 ± 0.844	506.71 ± 1.999
Mixed	8396	34.52 ± 0.574	1.41 ± 0.241	35.93 ± 0.521
Other woody vegetation	88573	254.33 ± 2.021	– 1.27 ± 1.541	253.06 ± 1.306
Plantations	61354	293.69 ± 1.987	– 0.68 ± 0.651	293.01 ± 1.877
USA				
Broadleaved	529916	2849.44 ± 9.183	188.01 ± 6.277	3037.45 ± 6.703
Coniferous	959568	6069.88 ± 15.075	– 23.96 ± 7.608	6045.92 ± 13.015
Mixed	665106	1482.73 ± 7.573	– 34.70 ± 5.873	1448.03 ± 4.781
Other woody vegetation	2351583	1023.21 ± 11.507	– 95.23 ± 11.074	927.98 ± 3.125
Plantations	1084568	2919.96 ± 9.789	– 239.26 ± 7.460	2680.70 ± 6.338

Negative values denote net emissions, net sequestration otherwise. Also shown are the 2010 areas of each UNSEEA class.

## Discussion

### Implications of reference data sample to carbon accounting

The reference data for the carbon flux spatial predictions did not cover the entire environmental space, wherein 6–47% of the combined forests and other woody vegetation areas appeared to be under-sampled. In such a case, one recommendation is to consider limiting the predictions without the under-sampled areas^19^. However, incomplete carbon accounting in the context of UNSEEA is inadvisable^3^. Hence, the results in the accounting table of countries with LiDAR samples particularly mixed forest and other woody vegetation (Fig. S4) should be treated with caution. Moving forward, we elaborate on a series of recommendations in cases where the country data sample would hamper the application of the mapped carbon flux for UNSEEA carbon accounting:

1. Additional samples: If additional field samples are not feasible, synthetic samples can be integrated into the current sample^47^. As proof of concept to this recommendation, we show the implications of adding synthetic samples within the under-sampled areas in Fig. S7. The current under-sampled areas are significantly reduced after adding pseudo loss samples based on forest loss data^48^. Gradual changes in forests such as from degradation and regrowth need to be sampled as well. Samples provided by forest dynamics monitoring tools can be explored^49^. Adding synthetic samples also means that a highly gapped clustered sample (Brazil) can turn into moderately gapped clustered data. In any case, a more suitable cross-validation should be used instead of the conventional* k-fold* cross-validation. For instance, cross-validation weighted by sampling intensity is suitable when using moderately clustered samples^16^.
2. Substitute the mapped carbon flux with the carbon flux from global products: For UNSEEA classes that are not supported by country data such as mixed forests and non-forest woody areas in this study, the mapped carbon flux can be substituted by global products (e.g. CCI and WRI). Either one or an average of the global products can be the alternative input data. This holds the rationale of a remote sensing-based carbon accounting. As a pre-requisite, an assessment of the global product can be initiated even at country scales using FAO-Forest Resource Assessment (FRA) as reference^14,32^. A good agreement of the country carbon fluxes between the global product and the FRA would justify the use of the former for carbon accounting.
3. Caveats for UNSEEA classes: Certain UNSEEA classes with carbon accounts that are highly uncertain such as mixed forests and other woody vegetation in our case, should have caveats for adoption and applications. This should be the case for both the physical carbon accounts (this study) and any subsequent monetary accounts. The caveats should be clear in the metadata and included in the data description of online data platforms (see Outlooks section).
4. Estimation of the uncertainty from sampling variability: Quantification of the uncertainty from sampling variability alongside mapping the under-sampled areas is our last recommendation. This sampling uncertainty can be estimated by bootstrapping methods that derive pairwise covariances of predicted carbon fluxes^50^. This step, however, may be applicable only for probability-based samples. Moreover, the procedure is computationally demanding and was demonstrated only at local scales from one study^11^. Aside from easing computational demands for country scales, worth testing is the quantification of sampling uncertainty with and without the pseudo samples.

### Accuracy and uncertainty of the spatial models

The predictive performance of our spatial models yielded: RMSE=9–39 Mg C $\textrm{ha}^{-1}$, ME=− 0.3–0.2 Mg C $\textrm{ha}^{-1}$ and $\textrm{R}^{2}$=0.16–0.71. These results are relatively higher when compared to our previous assessment of the biomass changes from global data ($\textrm{R}^{2}$=0.09-0.21)^14^. Nevertheless, mapping the changes in biomass and carbon using remote sensing is challenging. Pixel-level changes except for abrupt losses from deforestation can be very uncertain. For instance, the map producers of CCI maps cautioned about the use of their 100 m biomass change product due to uncertainties attributed to gradual changes in forests^22^. Similarly, Santoro et al. 2022^51^ found at least 40% relative uncertainty of biomass change in Sweden at 20 m pixel size. This map evaluation similarly used NFI as reference but their mapping used the indirect method of deriving biomass change by differencing two maps. Indirect methods are known to add up model errors from two epochs. Moreover, environmental dynamic inputs useful for direct carbon flux mapping were unavailable until 2021. Recent efforts already estimated carbon fluxes with high precision using direct methods. Esteban et al.^11^ for instance produced wall-to-wall growing stock volume (convertible to biomass) from combined NFI and airborne LiDAR as auxiliary data. McRoberts et al. 2022^50^ used a similar approach to estimate uncertainties from both residual and sampling variability intended for large-scale biomass mapping. More studies like this are anticipated considering the increasing number of permanent LiDAR sites as support for upcoming satellite missions^52^. This will strengthen our carbon accounting demonstration, where inputs are leaning towards time series data from airborne LiDAR^53^ and NFIs^54^. Such available datasets will allow annual carbon and carbon flux mapping. Annual maps can reveal trends in the carbon fluxes attributed to gradual changes. Additional base learners may also help improve the current ensemble model. This is important given that the predictions of base learners can be very different depending on how country data is sampled, as demonstrated in Fig. S5. Lastly, the prediction uncertainty can be assessed for example by means of the prediction interval e.g. accuracy plots^55^.

At pixel-level, LiDAR matches the spatial resolution (1 ha) of the remote sensing inputs (mostly 1 ha) used to predict carbon flux and this is in contrast to using NFIs where plots are 0.03–0.04 ha. The spatial mismatch when using NFIs may also be worsened by geo-location errors of plots especially in the tropics.

### Uncertainty sources of the carbon accounts

The carbon accounting required aggregation from pixel to UNSEEA class of carbon stocks (from CCI Biomass 2010) and fluxes. From the variograms in Fig. S6, we found a short-range correlation (<5 km) at country scales, which is consistent with previous studies particularly in Brazil^56^ and USA^57^. This basis allowed us to ignore spatial correlation during the aggregation step. This decision is case-to-case basis and needs reconsideration especially when the target country is relatively small i.e. where spatially correlated errors of around 5-50 km are already non-negligible. The variogram analysis also revealed the magnitude of autocorrelated scaled residuals (0–1) were all below 1. This suggests that we overestimate the map prediction error. This caution was also evident in 3 out of 4 global biomass maps in a previous study^28^. Here we focused on quantifying the uncertainty from residual variability, but missed to quantify the uncertainty from sampling variability (see 4th recommendation above). Another uncertainty source is the land cover input. Here we used the Level 2 UNSEEA classes but once countries require Levels 3 UNSEEA classes where, for instance, broadleaved is sub-classified into broadleaved closed canopy and broadleaved open canopy (Table S3), the uncertainty from land cover inputs needs consideration. Mapping more forest classes leads to a higher misclassification tendency among sub-classes. To account for this, land cover probabilities and geostatistical approaches are needed for potential area corrections among land cover units^58^. This correction also concerns the carbon accounting table where emissions and removals are reported separately and emission sources are disaggregated (Table S4). Should countries require such a table, the spatial dependency between emissions and removals needs to be accounted for as well. We finally emphasize that any reference data have a degree of uncertainty. We account for this uncertainty when we applied a weighted bootstrapping in the ensemble model that preferred samples with low measurement error uncertainty. An exactly similar approach was implemented by Araza et al.^28^ and Takoutsing and Heuvelink^55^. The latter example found no striking difference between a random forest model with and without the weighted bootstrapping.

### Influence of the covariates

The most influential covariates in the carbon flux predictions were global biomass products, which accounted for 51–70% of the overall importance scores of covariates. The carbon fluxes from CCI and WRI contributed the most to this score. This result seems to be expected and self-explanatory. What is more surprising is that we learned that other dynamic environmental datasets were also important covariates. Particularly, height dynamics was important for the spatial models of the Netherlands (62%) and USA (39%) as well as the $1\mathrm{{st}}$ and $2\mathrm{{nd}}$ NDVI quarterly composites for the European countries with at most 23% importance score. Height and vegetation indices are commonly used variables for biomass mapping because of their correlation to biomass^5^. To our knowledge, this is the first attempt to use their dynamic variables to model biomass change (the dynamic height dataset is relatively new). Moreover, elevation also had high importance score for Brazil (40%) and the Philippines (37%). This suggests that biomass change can be topography-driven in the tropics. The Brazil results need to be reanalyzed once more representative reference data becomes available, including for under-sampled biomes (such as Cerrado), remote locations with primary forests and mountainous areas with high elevation and slope. We found that the current sample in Brazil only concerns three out of the six topographic strata. Mountainous forested areas can also be prone to deforestation caused by landslides especially in typhoon season^6^. Recent studies revealed that biomass change in the tropics is seasonal^59^. Aside from including seasonal covariates, we recommend exploring other descriptive analysis of covariates to biomass change such as the use of partial dependence plots. These would show and visualize the marginal effects of the covariates on biomass change e.g. high biomass loss and low elevation.

### Country carbon fluxes

Overall, the carbon accounting tables depicted net carbon sequestration in natural forests except for Brazil, while net carbon emission was mostly observed in other woody vegetation and plantations (except for the Netherlands). Most net emissions were driven by land-use changes, while minimal emissions were depicted within forests i.e. forest degradation. The latter seems underestimated mainly because we used 100–300 m land cover inputs. An option is to replace them with 10 m global land cover data as long as the forest classes are consistent with UNSEEA. The net emissions within forests can be attributed to plantation activities such as thinning, salvage cutting and selective logging and activities that result in forest degradation such as timber poaching. Both emissions from forest conversion and forest degradation can be caused by environmental hazards such as strong winds, insects, landslides and fire.

As a good practice, we summarized the carbon fluxes and forest areas at country scales and compared them to similar results in the literature, see Table S5. In comparison with the FRA and other similar studies^21,22,33,51^, our estimates mostly depicted (agreed with 2 out of 3 sources) the Netherlands, the Philippines and Sweden as carbon sinks. In these countries, our estimates were often in between the estimates of FRA and similar studies suggesting we conservatively estimated carbon fluxes. While there is a large discrepancy in forest areas (since we included other woody non-forests) and a few years difference in the monitoring period, the comparisons in the results of Brazil and USA showed we overestimated the country carbon emissions compared to other sources. Recall that the samples for these countries are insufficient (see Fig. 3) which may allow the prediction models to overfit especially for a random cross-validation (Table 3). Our next carbon flux maps of the two countries will benefit from the anticipated USA Forest Inventory Analysis plots and additional re-measured LiDAR data for both USA and Brazil. Nevertheless, our estimates included reported uncertainties at the country level among the other sources. We also recommend further country cases based on the need to diversify the ecological conditions of case countries.

### Outlooks in carbon accounting based on remote sensing

Data sources of carbon flux maps are expected to further increase given the upcoming Earth Observation (EO) missions for forest monitoring. The development of EO-based data sources is seen as  an opportunity for the next generation UNSEEA accounts^9^. This also transpired in the last Advancing Earth Observation for Ecosystem Accounting conference in December 2022 https://eo4ea-2022.esa.int/. Remote sensing is also central to various projects that aim to upscale UNSEEA carbon accounting such as the following:

1. Pioneering Earth Observation Applications for the Environment (PEOPLE, https://esa-people-ea.org/en)
2. Artificial Intelligence for Environment and Sustainability (ARIES, aries.integratedmodelling.org)
3. Open Earth Monitor Project (OEMP, https://earthmonitor.org/)

Our current carbon accounting spans 8 years due to data availability for 2010 and 2018, but the UNSEEA ecosystem accounts are generally considered most useful if compiled at higher temporal resolution, e.g. annually or every 3 years^3^. We also plan to compile accounts for all carbon pools including at least the below-ground and soil components. In the future, increasing availability of satellite-derived biomass and carbon projects should allow this much higher temporal resolution of EO-based carbon accounts.

Carbon stocks and fluxes maps are always subject to map assessments and integration with reference data especially now that country NFIs are increasing even in developing countries in the tropics^54^. Given these country data in addition to the upcoming LiDAR sites^52^, sufficient global data would allow map errors of the global carbon fluxes to be modelled and minimized using our bias modelling approach^28^. A bias-adjusted global carbon flux welcomes the possibility of carbon accounting for all countries. This would not only benefit UNSEEA carbon accounting, but also the UNFCCC GHG reporting and even the UNFCCC Global Stocktake.

## Conclusions

To provide the carbon stock and flux input data required for UNSEEA carbon accounting, we spatially predict the 2010-2018 net carbon flux using ensemble machine learning in five countries with reference data. We found that mapping carbon fluxes at high resolution is challenging, judging by the variability in map accuracy results. Remote sensing is able to detect clear-cutting of forests and forest loss due to land conversion, but detecting gradual forest changes is more challenging. A further challenge in using remote sensing to estimate carbon fluxes is the saturation effect, which can be reduced with bias correction approaches^28,59^. Whereas there are clear uncertainties in pixel-level estimates of carbon fluxes derived from EO, these uncertainties are much reduced when pixels are aggregated to UNSEEA ecosystem types where carbon stocks and flows are reported by extent type, e.g. forest (Level 1) or deciduous forest (Level 2). For Level 2 classification, we found a short-range correlation of carbon flux map errors that can be considered negligible when we aggregate the carbon flux from pixel to UNSEEA.

The resulting carbon accounting tables revealed the net carbon sequestration in natural broadleaved forests. Both in plantations and in other woody vegetation ecosystems, emissions exceeded sequestration. Overall, our estimates align with FAO-Forest Resource Assessment and national studies with the largest deviations in Brazil and USA. These two countries used highly clustered reference data, where clustering caused uncertainty given the need to extrapolate to under-sampled areas. We anticipate that, with more EO data and reference data becoming available in the near future, annual carbon mapping will be increasingly feasible and can reveal trends concerning gradual forest changes. This allows for compiling more accurate, timely and cost-effective carbon accounts in line with UNSEEA.

### Supplementary Information

Supplementary Information.

## Data Availability

The contact person for the NFI reference data is Mart-Jan Schelhaas (martjan.schelhaas@wur.nl). Refer to the Labriere et al. 2018 paper for more information about the LiDAR datasets. The spatial models and information about the remote sensing datasets can be accessed here: https://github.com/arnanaraza/SEEA_RS.
