# Supplementary material for: Spatial predictions and uncertainties of forest carbon fluxes for carbon accounting
Source: Sci Rep. 2023 Aug 5;13:12704. doi: 10.1038/s41598-023-38935-8 (PMC10404296; doi:10.1038/s41598-023-38935-8)
Supplement: Supplementary file 1 — Supplementary Information. [file 41598_2023_38935_MOESM1_ESM.pdf]

Supplementary materials: Spatial predictions and uncertainties of forest carbon fluxes for environmental-economic accounting

Table S1. Reference data technical description

Country	Data type	Dominant forest type	Measurement (n)	Count (n)	Inventory year	Size (ha)	Mean AGB (Mg ha^{-1})	SD estimate	Eco-region
Brazil[1]	LiDAR	Natural	2	28607	2011-2018	1	-17.8	yes	Tropical rainforest
Netherlands[2]	NFI	Plantation	3	1562	2007-2016	0.04	11.8	no	Temperate broadleaf and mixed forests
Philippines[3]	NFI	Natural	2	587	2003-2014	0.5	2.8	yes	Tropical rainforest
Sweden[2]	NFI	Plantation	3	12887	2008-2013	0.03	4.9	no	Temperate broadleaf and Boreal forests
USA[4]	LiDAR	Natural	2	110939	2013-2019	1	1.76	yes	Temperate broadleaf and Boreal forests

Figure S1. Sample locations to highlight plot configurations of reference data.

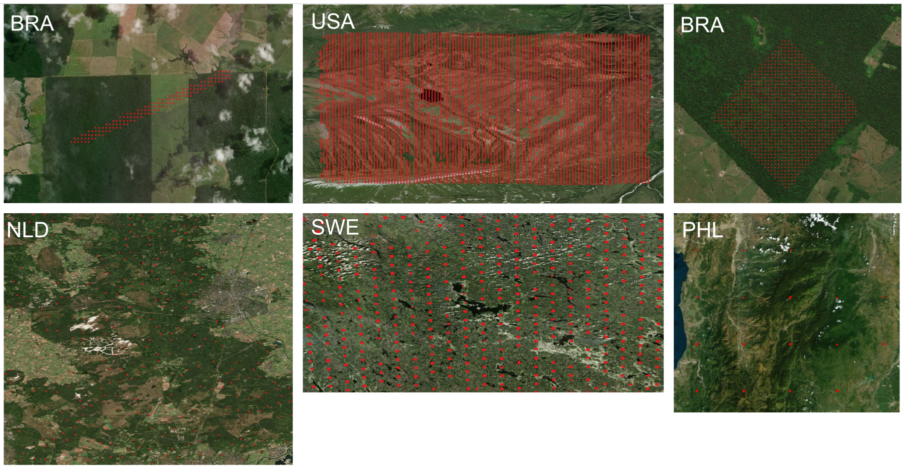

Table S2. Hyperparameter tuning results

Country	Model	Final parameters
BRA	SVM	C=0.2546
	XGM	nrounds=2000, max_depth=5, eta=0.03, gamma=0.01, colsample_bytree=0.75, min_child_weight=5, subsample=0.5
	RFM	min.node.size=5, mtry=18
NLD	SVM	C=0.0523
	XGM	nrounds = 500, max_depth = 5, eta = 0.01, gamma = 0.5, colsample_bytree = 0.75, min_child_weight = 10, subsample = 0.5
	RFM	min.node.size=5, mtry=2
PHL	SVM	C=0.0314
	XGM	nrounds=500, max_depth=5, eta=0.01, gamma=0.5, colsample_bytree=0.75, min_child_weight=1, subsample=0.5
	RFM	min.node.size=5, mtry=2
SWE	SVM	C=15.032
	XGM	nrounds = 500, max_depth = 5, eta = 0.01, gamma = 0.1, colsample_bytree = 0.75, min_child_weight = 10, subsample = 0.5
	RFM	min.node.size=5, mtry=12
USA	SVM	C=2.864
	XGM	nrounds=2000, max_depth=5, eta=0.1, gamma=0.01, colsample_bytree=0.75, min_child_weight=1, subsample=0.5
	RFM	min.node.size=5, mtry=10

Other base learners

We used extreme gradient boosting models[5] (XGM) as base learner. Like other boosting models, XGM is capable of extrapolating beyond the range of the training data i.e., the under-sampled areas in our case (see Figure S4). This boosting model also grows decision trees sequentially and create an ensemble after final predictions. Unlike other boosting models, XGM can optimize how trees are grown by applying weighted decision trees as tree pruning basis. Moreover, XGM extends the optimization of an objective function from first-order to second-order Taylor expansion. The objective function (i.e., loss gradient) is the optimization metric that should be as minimized as possible after model fitting. The model can also perform parallel computing, data imputation and, like RFM, minimizes model overfitting but using a regularization term instead of decorrelating trees. Another base learner is support vector machine[6] (SVM), an algorithm that tries to find the line or boundary (hyperplane) that best fits the data. Instead of separating two classes like in classification, the SVM is trying to fit the data optimally. The SVM tries to find the hyperplane that minimizes the distance between the data points and the hyperplane, while also maximizing the distance between the hyperplane and the nearest data points. The model is controlled by a regularization term (C) or a trade-off between the complexity of the model and the amount of training error that is tolerated.

Figure S2. Forest classes of each country. We used QGIS 3.4.3[7] to layout this map.

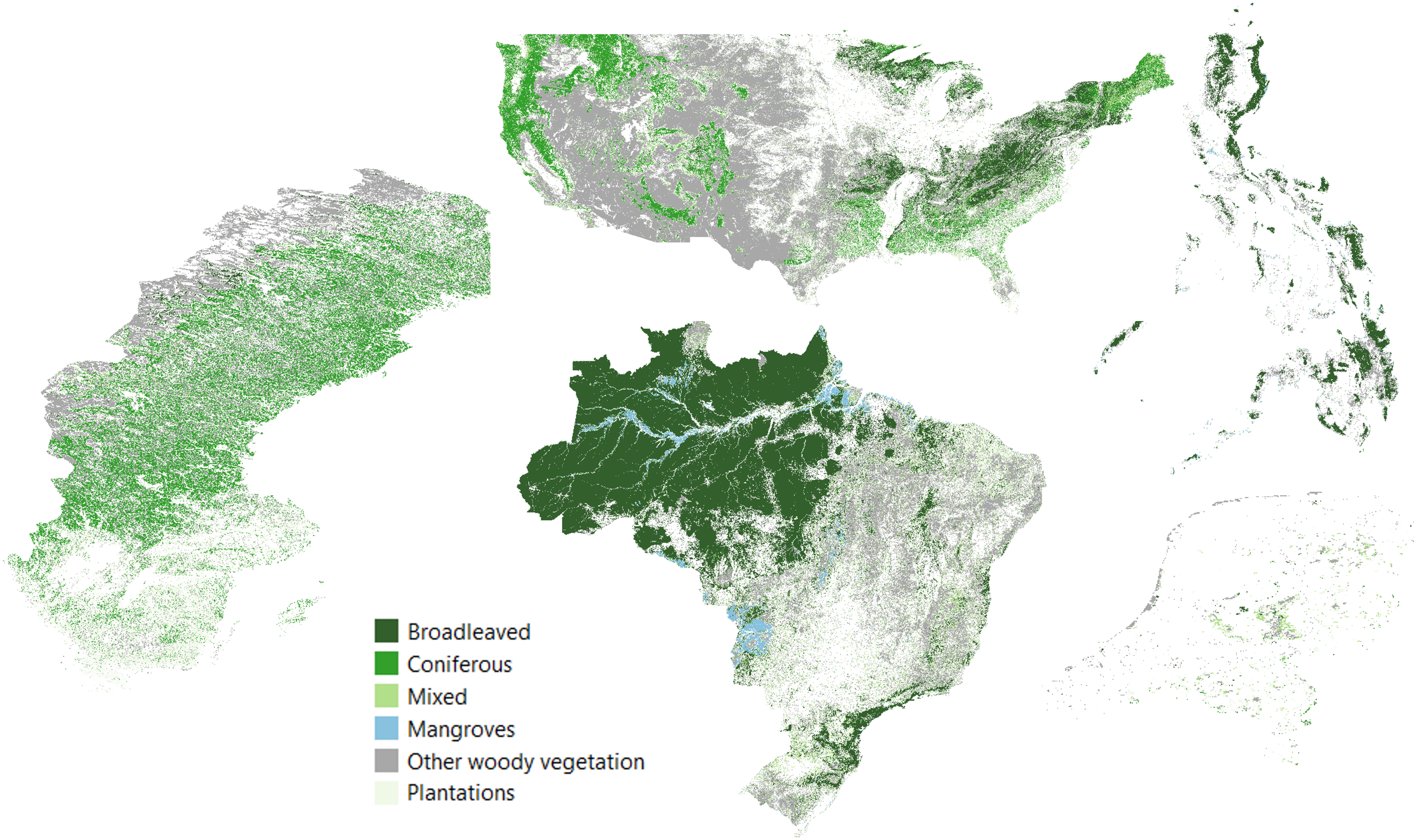

Table S3. Reclassification of land cover datasets into Level 1 UN-SEEA classes. Note that the 2nd column denotes the actual class number of the land cover dataset.

CCI Land Cover[8]	50 = Tree cover, broadleaved, evergreen, closed to open (>15%)	1 = Broadleaved forest
	70-72 = Tree cover, needleleaved, evergreen, closed to open (>15%)	2 = Coniferous forest
	90 = Tree cover, mixed leaf type (broadleaved and needleleaved)	3 = Mixed forest
	100 = Mosaic tree and shrub (>50%) / herbaceous cover (<50%)	4 = Mangroves
	160 = Tree cover, flooded, fresh or brackish water	
	170 = Tree cover, flooded, saline water	
	180 = Shrub or herbaceous cover, flooded, fresh/saline/brakish water	
CORINE[9]	23 = Broadleaved forest	1 = Broadleaved forest
	24 = Coniferous forest	2 = Coniferous forest
	25 = Mixed forest	3 = Mixed forest
	26 = Natural grassland	5 = Other woody vegetation
IIASA data[10]	31 = Planted forests (rotation >15 years)	6 = Forest plantation
	32 = Plantation forest (rotation <15 years)	
	40 = Oil palm plantations years	
	53 = Agroforestry	

Figure S3. Correlation matrix of covariates for the five case countries.

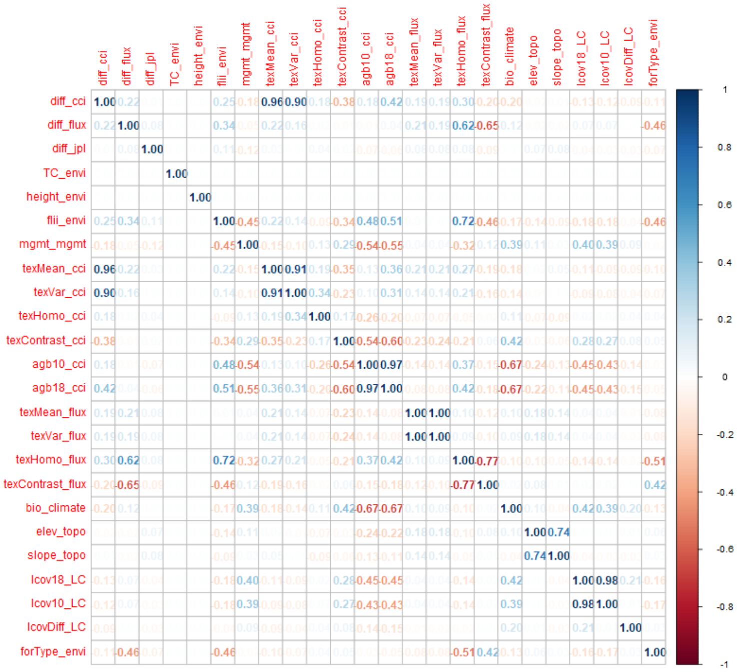

Brazil

Residual spatial correlation modelling

The resulting carbon flux map from the spatial predictions were multiplied by 0.49 to derive carbon fluxes. The carbon flux map residuals $MR(i)$, defined as map-reference data carbon flux difference at plot location i, was scaled by the map SD (SD_{m}); this assumes the SD of the residuals is proportional to the SD_{m} at that point (equation 1). This scaling was assumed to transform the residuals to homoscedasticity $SR(x)$. For carbon stocks, we used the existing SD layer of CCI 2010 multiplied by 0.49.

$$SR(x) = \frac{MR(x)}{SD_m(x)} \quad (1)$$

Models of semivariances, $\gamma(h)$ in equation 2, allow estimation of the spatial correlation of SR at spatial lag h, where x is a plot location, and the errors are assumed to be statistically stationary:

$$\gamma(h) = \frac{1}{2}\mathrm{Var}[SR(x) - SR(x+h)] \quad (2)$$

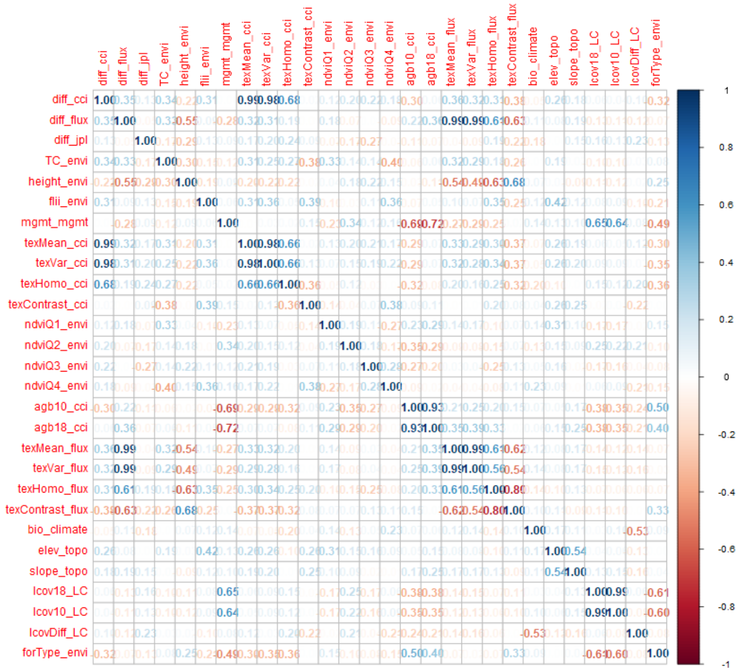

Netherlands

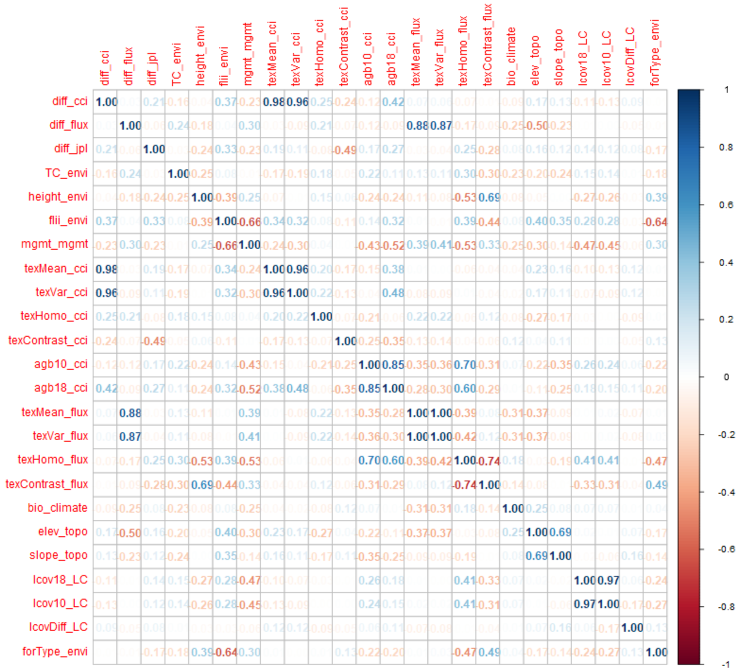

Philippines

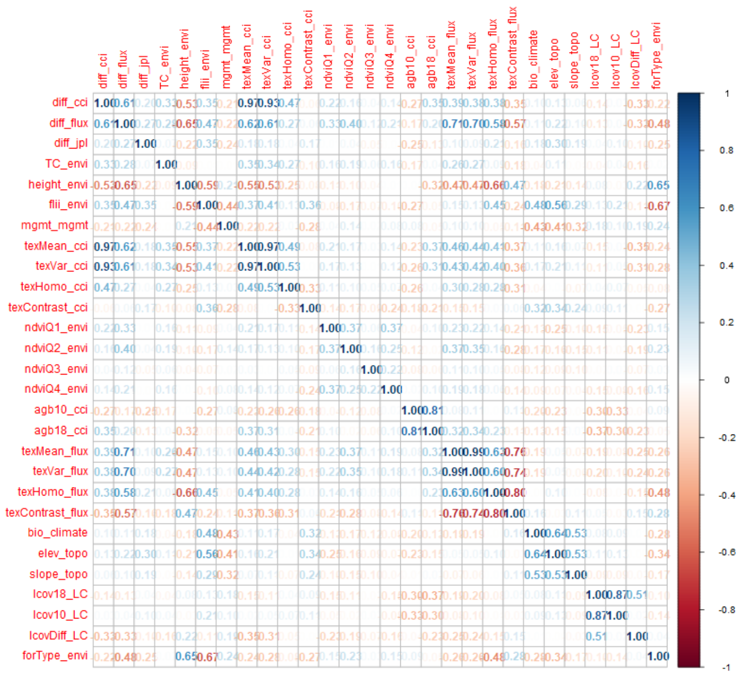
diff_cci
diff_flux
diff_jpl
TC_envi
height_envi
flii_envi
mgmt_mgmt
texMean_cci
texVar_cci
texHomo_cci
texContrast_cci
ndviQ1_envi
ndviQ2_envi
ndviQ3_envi
ndviQ4_envi
agb10_cci
agb18_cci
texMean_flux
texVar_flux
texHomo_flux
texContrast_flux
bio_climate
elev_topo
slope_topo
lcov18_LC
lcov10_LC
lcovDiff_LC
forType_envi
1
0.8
0.6
0.4
0.2
0
-0.2
-0.4
-0.6
-0.8
-1

Sweden

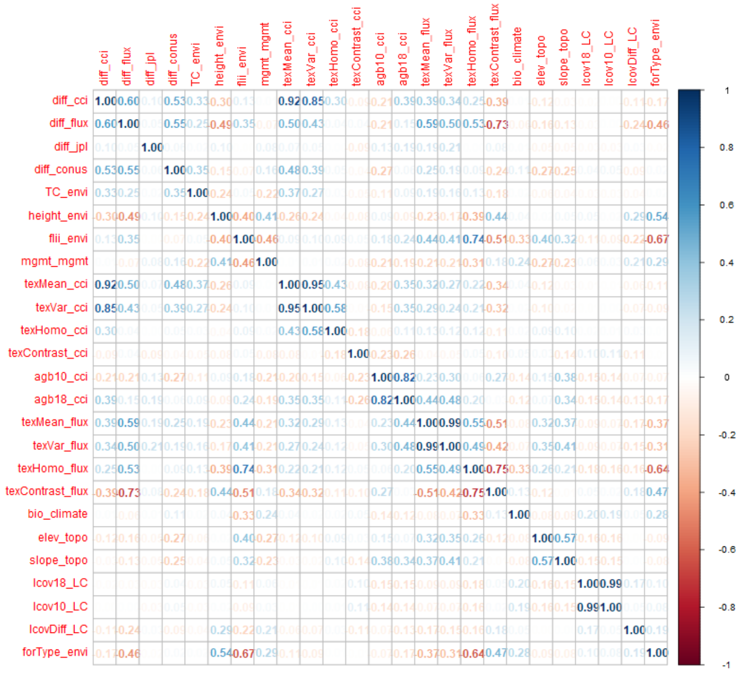
diff_cci
diff_flux
diff_jpl
diff_conus
TC_envi
height_envi
flii_envi
mgmt_mgmt
texMean_cci
texVar_cci
texHomo_cci
texContrast_cci
agb10_cci
agb18_cci
texMean_flux
texVar_flux
texHomo_flux
texContrast_flux
bio_climate
elev_topo
slope_topo
lcov18_LC
lcov10_LC
lcovDiff_LC
forType_envi
1
0.8
0.6
0.4
0.2
0
-0.2
-0.4
-0.6
-0.8
-1

USA

Figure S4. Proportion of under-sampled areas per UN-SEEA carbon accounting classes for each country

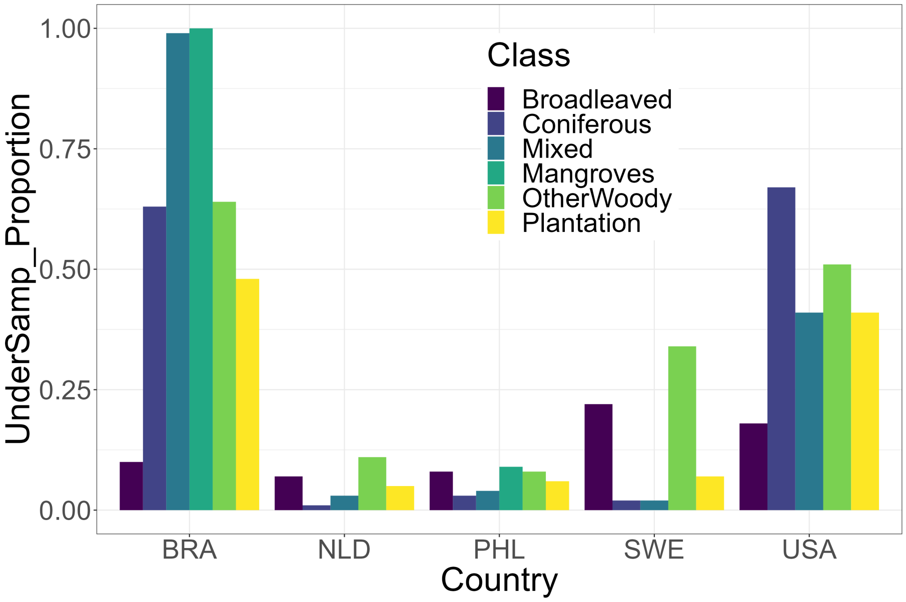

Figure S5. Prediction comparison of random forest and extreme gradient boosting when using systematic and preferential samples.

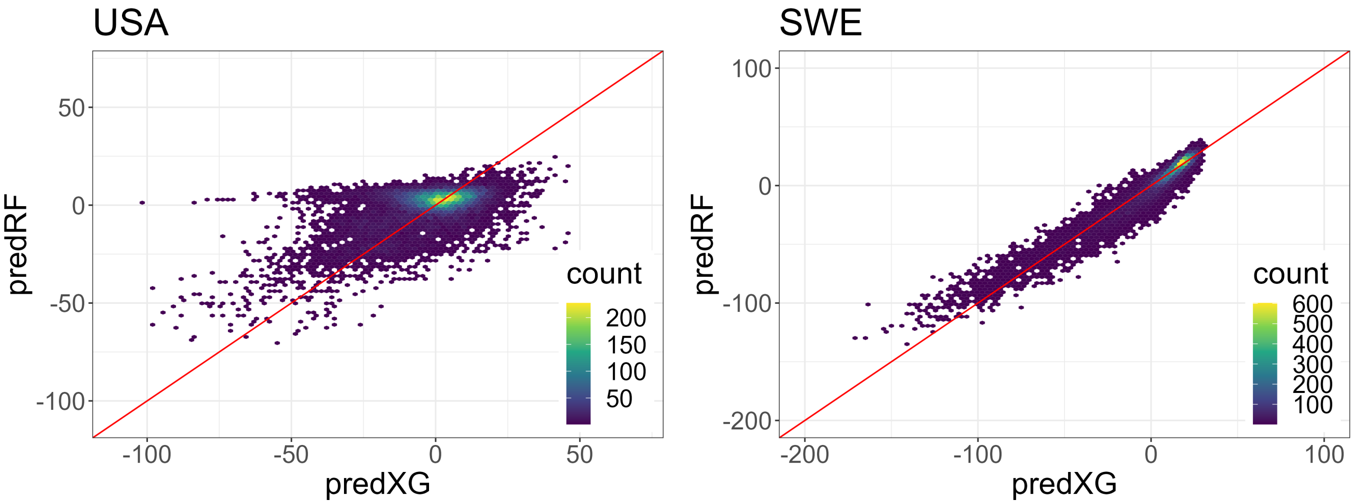

Figure S6. Variograms of stocks and flow residuals.

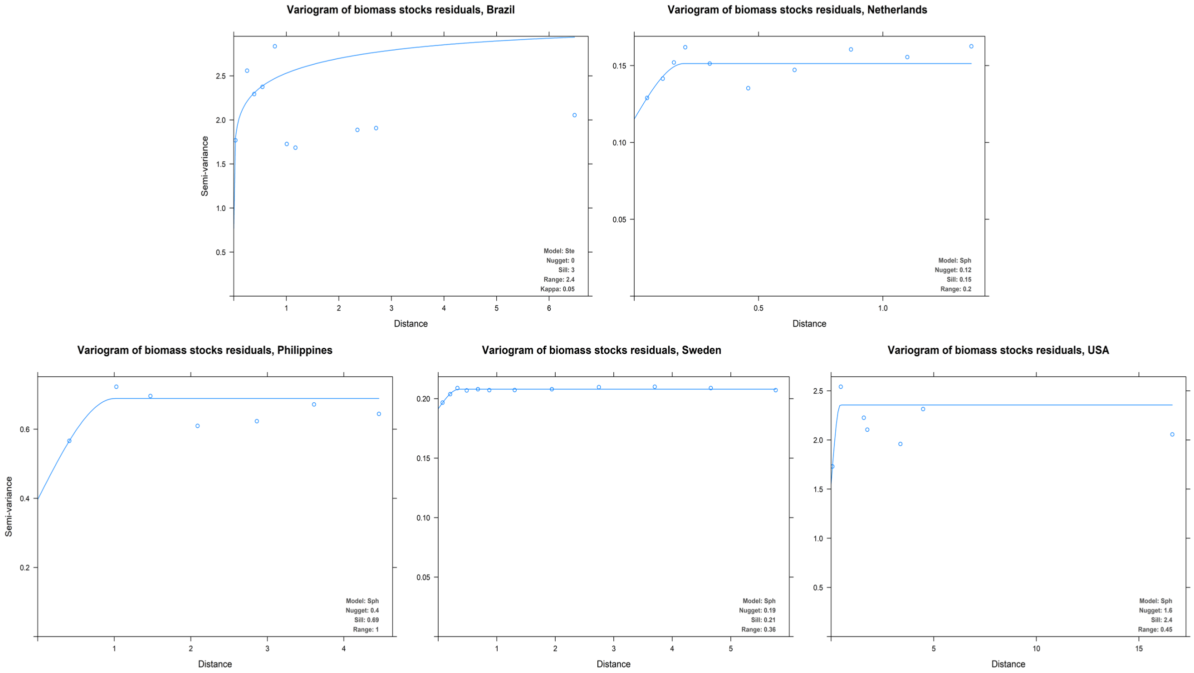

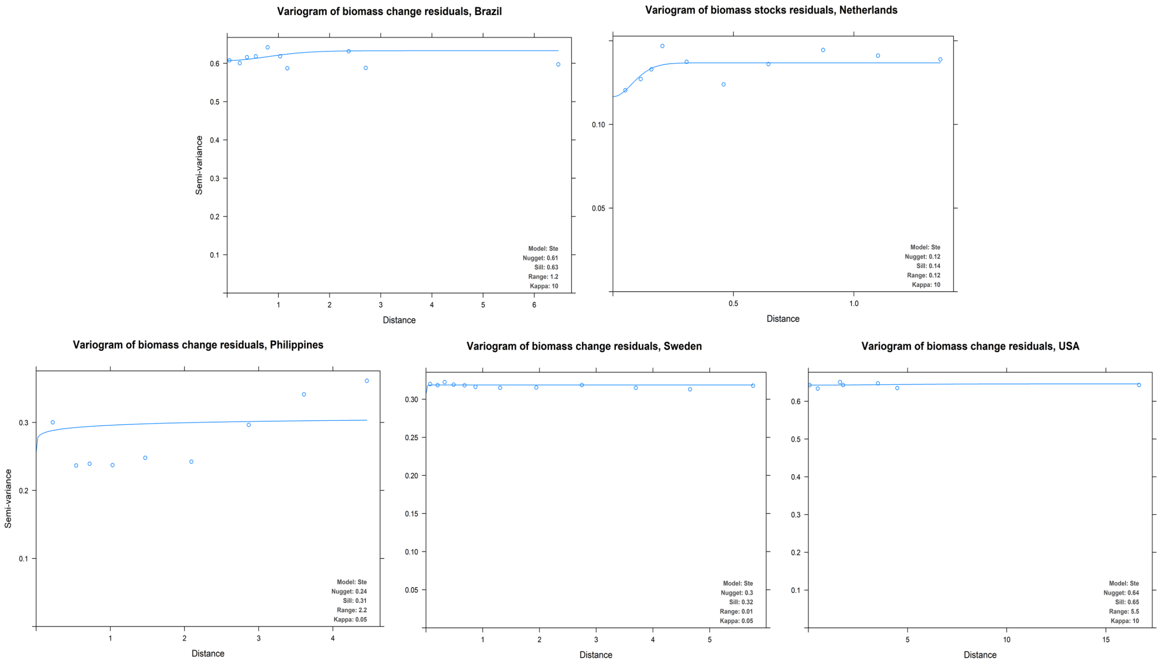
Variogram of biomass change residuals, Brazil
Semi-variance
Distance
Model: Ste
Nugget: 0.61
Sill: 0.63
Range: 1.2
Kappa: 10
Variogram of biomass stocks residuals, Netherlands
Distance
Model: Ste
Nugget: 0.12
Sill: 0.14
Range: 0.12
Kappa: 10
Variogram of biomass change residuals, Philippines
Semi-variance
Distance
Model: Ste
Nugget: 0.24
Sill: 0.31
Range: 2.2
Kappa: 0.05
Variogram of biomass change residuals, Sweden
Distance
Model: Ste
Nugget: 0.3
Sill: 0.32
Range: 0.01
Kappa: 0.05
Variogram of biomass change residuals, USA
Distance
Model: Ste
Nugget: 0.64
Sill: 0.65
Range: 5.5
Kappa: 10

Figure S7. Impact of pseudo loss data to the under-sampled areas(a) with and (b) without pseudo loss samples. The randomly selected pseudo samples are limited to 10% of the total country reference data and sampled within the under-sampled areas in (b). We used *ggplot2* in R[11] to layout this map.

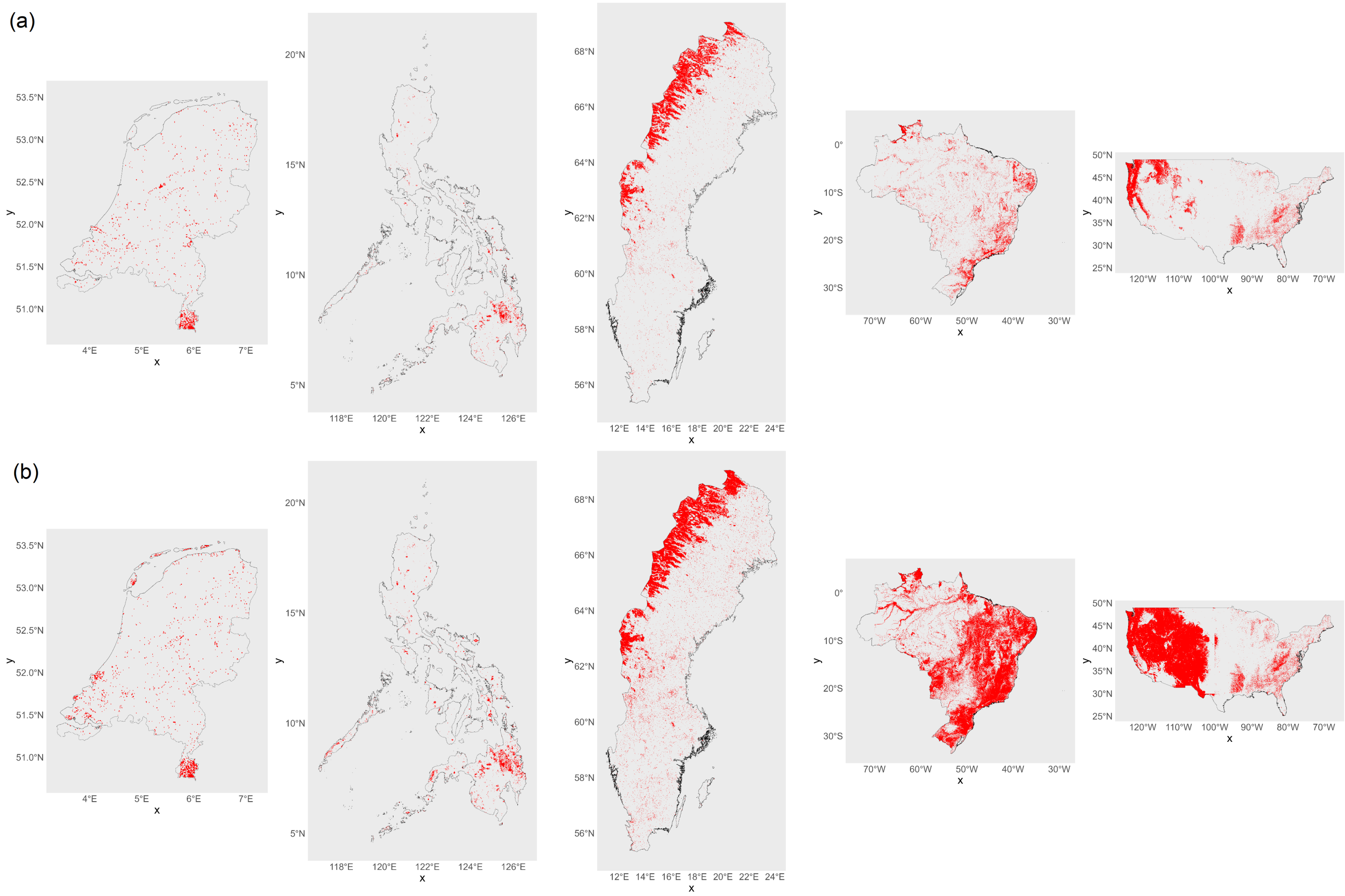

Table S4. Carbon accounting table with emissions and removals. The emissions are further disaggregated whether as a result of land use change (forest conversion) of emissions within forest (likely forest degradation). Aside from the 2010 land cover dataset, we used the associated 2018 land cover for this disaggregations.

Countries	UN-SEEA classes	Forest area 2010 ('000 ha)	Net emissions (C Mg ha $^{-1}$)	Emissions from land-use change (C Mg ha $^{-1}$)	% contribution	Emissions within forest (C Mg ha $^{-1}$)	% contribution	Net sequestration (C Mg ha $^{-1}$)	Net fluxes (C Mg ha $^{-1}$)
Brazil	Broadleaved	3485634	-127.43	-127.26	99.87%	-0.17	0.13%	84.25	-43.18
	Coniferous	153277	-3543.74	-3543.74	100.00%	0.00	0.00%	3520.98	-22.76
	Mixed	735145	-479.66	-479.66	100.00%	0.00	0.00%	323.64	-156.02
	Mangroves	166677	-1.81	-1.79	98.90%	-0.02	1.10%	1.33	-0.48
	Other woody vegetation	1140095	-673.62	-664.73	98.68%	-8.88	1.32%	270.12	-403.50
	Plantation	1320002	-738.59	-728.48	98.63%	-10.10	1.37%	317.41	-421.18
Netherlands	Broadleaved	89	-0.002	0.00	98.78%	0.00	1.22%	0.01	0.002
	Coniferous	103	-0.001	0.00	98.97%	0.00	1.03%	0.001	0.001
	Mixed	530	-0.002	0.00	99.09%	0.00	0.91%	0.02	0.02
	Other woody vegetation	1109	-0.21	-0.20	98.98%	0.00	0.00%	0.23	0.02
	Plantation	1206	-0.02	-0.01	98.96%	0.00	1.04%	0.04	0.02
Philippines	Broadleaved	57334	-4.95	-4.90	99.09%	0.00	0.00%	8.81	3.87
	Coniferous	3008	-0.21	-0.21	98.93%	0.00	0.00%	0.35	0.14
	Mixed	4962	-0.80	-0.77	95.23%	0.00	0.00%	0.52	-0.28
	Mangroves	5487	-2.58	-2.50	96.88%	0.00	0.00%	1.48	-1.10
	Other woody vegetation	54494	-5.48	-5.48	100.00%	0.00	0.00%	7.31	1.83
	Plantation	15199	-4.91	-4.46	90.90%	-0.45	9.10%	4.05	-0.86
Sweden	Broadleaved	4588	-0.04	-0.04	98.46%	0.00	1.54%	0.36	0.32
	Coniferous	109355	-7.19	-7.11	98.85%	-0.08	1.15%	12.78	5.59
	Mixed	8396	-0.40	-0.40	100.00%	0.00	0.00%	1.81	1.41
	Other woody vegetation	88573	-17.80	-17.76	99.80%	-0.04	0.20%	16.53	-1.27
	Plantation	61354	-5.04	-4.95	98.11%	-0.10	1.89%	4.36	-0.68
USA	Broadleaved	529916	-61.89	-59.60	96.29%	-2.30	3.71%	249.90	188.01
	Coniferous	959568	-418.90	-398.79	95.20%	-20.12	4.80%	394.94	-23.96
	Mixed	665106	-161.11	-161.11	100.00%	0.00	0.00%	126.41	-34.70
	Other woody vegetation	2351583	-292.89	-276.10	94.27%	0.00	0.00%	197.65	-95.23
	Plantation	1084568	-420.88	-408.62	97.09%	-12.26	2.91%	181.62	-239.26

Table S5. Summary of country net carbon fluxes 2010-2018 and inter-comparison with other sources. Reported also are the 2010 forest area and the % change of the 2010 stocks relative to the 2010-2018 fluxes.

Country	Area ('000 km^2)	C Flux (Tg)	% change
BRA			
This study	7000830	-1,047.12	-0.015%
CCI	7000830	2,173.22	0.031%
WRI	7000830	599.80	0.009%
FRA	5115810	-435.6	-0.085%
NLD			
This study	3037	0.06	0.002%
CCI	3037	-0.11	-0.004%
WRI	3037	0.41	0.014%
FRA	3735	0.911	0.024%
PHL			
This study	125285	4.46	0.004%
CCI	125285	51.23	0.041%
WRI	125285	50.98	0.041%
FRA	68397	-4.03	-0.006%
SWE			
This study	272266	5.37	0.002%
CCI	272266	-30.71	-0.011%
WRI	272266	618.25	0.227%
FRA	280730	37.65	0.013%
Santoro 2010-2015[12]	254646	12	0.005%
USA			
This study	5590741	-205.14	-0.004%
CCI	5590741	-30.71	-0.001%
WRI	5590741	618.25	0.011%
FRA	3087200	644.67	0.021%
CONUS 2010-2017[13]	4313653	224.5	0.005%

References

1. Longo, M. *et al.* Aboveground biomass variability across intact and degraded forests in the brazilian amazon. *Glob. Biogeochem. Cycles* **30**, 1639–1660, DOI: 10.1002/2016gb005465 (2016).
2. Schelhaas, M.-J. *et al.* Actual european forest management by region, tree species and owner based on 714, 000 re-measured trees in national forest inventories. *PLOS ONE* **13**, e0207151, DOI: 10.1371/journal.pone.0207151 (2018).
3. Araza, A., Herold, M., Hein, L. & Quinones, M. The first above-ground biomass map of the philippines produced using remote sensing and machine learning. In *2021 IEEE International Geoscience and Remote Sensing Symposium IGARSS*, DOI: 10.1109/igarss47720.2021.9553225 (IEEE, 2021).
4. Johnson, B. R., Kuester, M. A., Kampe, T. U. & Keller, M. National ecological observatory network (NEON) airborne remote measurements of vegetation canopy biochemistry and structure. In *2010 IEEE International Geoscience and Remote Sensing Symposium*, DOI: 10.1109/igarss.2010.5654121 (IEEE, 2010).
5. Chen, T. & Guestrin, C. XGBoost. In *Proceedings of the 22nd ACM SIGKDD International Conference on Knowledge Discovery and Data Mining*, DOI: 10.1145/2939672.2939785 (ACM, 2016).
6. Hearst, M. A., Dumais, S. T., Osuna, E., Platt, J. & Scholkopf, B. Support vector machines. *IEEE Intell. Syst. their applications* **13**, 18–28 (1998).
7. QGIS Development Team. *QGIS Geographic Information System*. QGIS Association (2023).
8. Defourny, P. *et al.* Land cover cci. *Prod. User Guid. Version* **2**, 325 (2012).
9. Büttner, G. CORINE land cover and land cover change products. In *Land Use and Land Cover Mapping in Europe*, 55–74, DOI: 10.1007/978-94-007-7969-3_5 (Springer Netherlands, 2014).
10. Lesiv, M. *et al.* Global forest management data for 2015 at a 100 m resolution. *Sci. Data* **9**, DOI: 10.1038/s41597-022-01332-3 (2022).
11. Wickham, H. *ggplot2: Elegant Graphics for Data Analysis* (Springer-Verlag New York, 2016).
12. Santoro, M., Cartus, O. & Fransson, J. E. Dynamics of the swedish forest carbon pool between 2010 and 2015 estimated from satellite l-band SAR observations. *Remote. Sens. Environ.* **270**, 112846, DOI: 10.1016/j.rse.2021.112846 (2022).
13. Yu, Y. *et al.* Making the US national forest inventory spatially contiguous and temporally consistent. *Environ. Res. Lett.* **17**, 065002, DOI: 10.1088/1748-9326/ac6b47 (2022).
